# Exploring Universal Domain Adaptation with CLIP Models: A Calibration Method

**DOI:** 10.3390/e27121213

**Published:** 2025-11-28

**Authors:** Bin Deng

**Affiliations:** School of Electronic and Information Engineering, Wuyi University, Jiangmen 529020, China; bindeng@wyu.edu.cn

**Keywords:** Universal Domain Adaptation, CLIP, model calibration

## Abstract

CLIP models have shown their impressive learning and transfer capabilities in a wide range of visual tasks. It is, however, interesting that these foundation models have not been fully explored for Universal Domain Adaptation (UniDA). In this paper, we make comprehensive empirical studies of state-of-the-art UniDA methods using these foundation models. We first demonstrate that although the foundation models greatly improve the performance of the baseline method (which trains the models on the source data alone), existing UniDA methods struggle to improve over the baseline. This suggests that new research efforts are necessary for UniDA using these foundation models. Finally, we observe that calibration of CLIP models plays a key role in UniDA. To this end, we propose a very simple calibration method via automatic temperature scaling, which significantly enhances the baseline’s out-of-class detection capability. We show that a single learned temperature outperforms previous approaches in most benchmark tasks when adapting from CLIP models, excelling in evaluation metrics including H-score and a newly proposed Universal Classification Rate (UCR) metric. We hope that our investigation and the proposed simple framework can serve as a strong baseline to facilitate future studies in this field.

## 1. Introduction

A foundational goal of machine vision is to develop a model that can be applied to data from different distributions. With the emergence of many large-scale pre-trained models such as CLIP [1], ALIGN [2], and DINOv2 [3], significant progress has been made recently toward achieving this goal. These “foundation models” [4] often exhibit significantly greater robustness to various benchmark distribution shifts compared to standardly trained models. For instance, in image classification, while both CLIP and a standard ImageNet-pretrained model attain an accuracy of 76% on ImageNet, CLIP exhibits notable enhancements, with an accuracy increase of 6% on ImageNetV2 and 35% on ImageNet Sketch [1]. Due to the powerful capabilities of these foundation models, techniques for applying them to downstream applications are increasingly important. Indeed, the research community has spent significant effort over the past few years on improving the fine-tuning of these models for various downstream tasks, including few-shot classification [5], out-of-distribution (OOD) detection [6], and OOD generalization [7,8], among others.

Interestingly, Universal Domain Adaptation (UniDA) [9]—a practical setting that aims to adapt a model to a specific target domain without restrictions on the label sets—has not been thoroughly explored to date using powerful foundation models. This paper aims to fill this gap by initially assessing the performance of state-of-the-art UniDA methods when applied to foundation models such as CLIP. Through comprehensive experiments, we present several interesting findings. As expected, all methods achieved substantial improvements over their prior results that relied on ImageNet pre-trained models. However, the performance gap between the Source-Only (SO) baseline and the state-of-the-art (SOTA) methods has notably narrowed, rendering them largely comparable across various benchmark tasks. These findings suggest that new research efforts are necessary for UniDA in the context of foundation models.

Finally, we recognize the pivotal role that the calibration of CLIP foundation models plays in UniDA. To this end, we introduce a simple calibration method by learning a single temperature parameter using source data. Our method, which requires no additional model training, significantly enhances the baseline’s capability to detect out-of-class instances. Despite its simplicity, our method demonstrates exceptional robustness and efficacy across various task scenarios, including open-partial, open, closed, and partial UniDA settings. It excels according to both the established H-score metric and the novel UCR metric. We hope that this straightforward approach can serve as a solid baseline for future research on UniDA using CLIP models.

The main contributions of this paper are summarized as follows:

- To the best of our knowledge, we are the first to tackle the UniDA problem and conduct a comprehensive study of existing methods when applied to CLIP models. Our findings underscore the urgent need for further research in UniDA using these powerful foundation models.
- We propose a straightforward calibration method for UniDA, establishing a new baseline for adaptation from CLIP models. Our approach incorporates a self-calibration technique based on automatic temperature scaling, making it parameter-free and robust across various task scenarios.
- We introduce a novel evaluation metric for UniDA, the Universal Classification Rate (UCR), which is insensitive to thresholds and class ratios. Additionally, to facilitate rigorous and replicable experimentation in UniDA, we have developed and made publicly available the UniOOD framework. UniOOD simplifies the incorporation of new datasets and algorithms with only a few lines of code, thereby ensuring fairer comparisons between different methods.

## 2. Related Works

**Domain Adaptation (DA).** A cornerstone of domain adaptation is marginal distribution alignment, commonly achieved through explicit discrepancy measures (e.g., MMD [10], covariance alignment [11]) or adversarial training, as exemplified by the Domain Adversarial Neural Network (DANN) [12]. Subsequent adversarial methods have sought to achieve finer conditional alignment by incorporating techniques like pseudo-labeling [13] or dual-classifier discriminators [14,15]. More recently, Adversarial Self-Training (AST) [16] was introduced, demonstrating notable gains in both adversarial and clean accuracy for gradual DA tasks. The success of Transformers in various tasks has also inspired their use in DA. For instance, the WinWin Transformer (WinTR) [17] exploits domain-specific and invariant knowledge through target pseudo-label refinement and contrastive learning. CDTrans [18] employs a triple-branch architecture with a cross-attention mechanism to align cross-domain image pairs via patch similarity. Building on an adversarial framework, SSRT [19] utilizes perturbed target predictions for safe self-refinement. Recently, DoT [20] further learned locality consistency and reduced generalization error through automated domain-level attention. Despite these advances, most existing DA methods are designed for and evaluated in the closed-set category setting.

**Universal Domain Adaptation.** Different from the traditional DA problem, which assumes all labels in the target domain are identical to the source domain, UniDA [9] assumes that there is no prior knowledge about the label relationship between source and target domains. Due to the existence of labels shift in UniDA, classical DA methods of adversarial adaptation such as DANN [12] often suffer from negative transfer. To address this problem, UAN [9] and CMU [21] use sample-level uncertainty criteria to assign weights for each sample before adversarial alignment. In addition to adversarial adaptation, self-training or self-supervised-based methods usually have better performance due to the exploiting of discriminative representation in the target domain. Among these, DANCE [22] uses self-supervised neighborhood clustering to learn the target data structure; DCC [23] exploits cross-domain consensus knowledge to discover discriminative clusters of both domains; MATHS [24] designs a contrastive learning scheme to nearest neighbors for feature alignment; OVANet [25] proposes to train a one-vs-all classifier for each class and applies entropy minimization to target samples during adaptation; and more recently, UniOT [26] uses optimal transport criteria to select more confident clusters to target samples for self-training. However, all of these methods are evaluated solely using models pre-trained in ImageNet. In this paper, we compare against the most state-of-the-art methods under the foundation models such as CLIP. We show that there exists a strong baseline that can be competitive with or outperform the more complex methods listed above when using these foundation models.

**Domain Generalization (DG).** Unlike DA, DG assumes no access to target domain data during training. To enhance model robustness, DG methods typically employ strategies such as data augmentation at the image level [27] or feature level [28], remove spurious correlations via stable learning [29], or leverage the inherent inductive bias of neural networks [30,31]. However, many of these methods are empirically driven and their effectiveness is often validated on specific benchmarks. Recent empirical studies [32,33] have raised concerns about the actual efficacy of numerous DG algorithms, suggesting that current benchmark-based evaluations may be inadequate. In contrast to typical DG approaches, our work deliberately refrains from applying any specialized DG techniques (e.g., data augmentation). This design choice allows us to isolate and investigate the true performance of UniDA methods when integrated with CLIP models.

**Adaptation of CLIP Models.** The exceptional performance of foundation models in traditional vision tasks has led to a growing interest in developing more effective adaptive methods. In addition to adopting linear probing [34], full fine-tuning [35], or zero shot [1] to the backbone models, many new strategies or methods have been proposed. For example, prompt learning based methods [36,37,38] propose to learn better prompts under the language-vision models. CLIP-Adapter [39] and Tip-Adapter [40] are going to construct additional light models for efficient fine-tuning while freezing the backbone models. Surgical fine-tuning [8] suggests selective fine-tuning of a subset of layers based on different types of distribution shift. WiSE-FT [7] proposes to enhance the model robustness by integrating the zero-shot model and the fine-tuning model. And more recently, cross-model adaptation [5] shows the most powerful few-shot ability to CLIP based models by incorporating multi-modalities as training samples for ensemble training. In this paper, different from all these methods that aim to adapt models for closed-set classification task, we exploit to adapt for UniDA problem. We also show how effective would be if these representative methods are directly applied for the UniDA tasks.

**Related Subfields.** UniDA is also closely related to Open-Set Recognition (OSR) [41] and OOD detection [42]. OSR extends the closed-set classification to a more realistic open-set classification, where test samples may come from domains of unknown classes. This setting is very similar to UniDA but it assumes that there exists no domain shift and that one can not access the target domain during training. OOD detection, on the other hand, focuses on detecting the out-of-class samples only. In theory, a recent work of [43] unifies OSR and OOD detection into the same framework and shows that the loss criterion must be carefully designed otherwise it may face an intractable learning problem. In this paper, we are inspired by these works and introduce a similar evaluation metric of UCR for UniDA. Although many methods of OSR and OOD detection have been proposed during the past few years, a recent empirical study by Vaze et al. [44] shows that a good closed-set classifier can be competitive with or even superior to previous complex methods. These findings align with our results on UniDA under the foundation models.

## 3. Problem Formulation

In UniDA, we are provided with a source domain dataset $D^{s}={\left\{\left(x_{i}^{s},y_{i}^{s}\right)\right\}}_{i=1}^{n_{s}}$ consisting of $n_{s}$ samples, where the *i*-th sample $x_{i}^{s}\in R^{d}$ is a *d* dimensional vector and $y_{i}^{s}\in Y^{s}$ is the associated label. Additionally, we have a target domain dataset $D^{t}={\left\{\left(x_{i}^{t}\right)\right\}}_{i=1}^{n_{t}}$, which contains $n_{t}$ unlabeled samples from the same *d*-dimensional space. Samples in the source and target domains are drawn from their respective distributions, $D^{s}∼\pi_{s}\left(X^{s},Y^{s}\right)$ and $D^{t}∼\pi_{t}\left(X^{t},Y^{t}\right)$. We represent the collection of labels in the source domain as $Y^{s}$ and in the target domain as $Y^{t}$. Let $Y^{st}=Y^{s}\cap Y^{t}$ be the domain-shared label set and $Y^{t/s}=Y^{t}\setminus Y^{s}$ be the target-private label set. Similarly, $Y^{s/t}$ is the set of source-private labels. In UniDA, we make no assumptions about $Y^{t}$. Hence, $Y^{st}$ and $Y^{t/s}$ are also unknown. For convenience, we refer to target samples belonging to $Y^{st}$ (known classes) as in-of-class samples $D_{in}^{t}$ and those belonging to $Y^{t/s}$ (unknown classes) as out-of-class samples $D_{out}^{t}$.

The learning task of UniDA can be converted as two subtasks of in-of-class discrimination and out-of-class detection. Such objectives could be implemented by a unified framework as: (1) learning a scoring function $s:R^{d}\to R$ for out-of-class detection and (2) learning a classifier $f:R^{d}\to R^{|Y^{s}|}$ for in-of-class discrimination. The scoring function *s* assigns a score to each sample, which reflects the uncertainty level regarding it being an out-of-class sample. A higher score indicates a higher likelihood of belonging to the in-of-class category. UniDA methods require a threshold value for the scoring function *s* to distinguish between out-of-class and in-of-class samples. This threshold can either be learned automatically or set manually.

Typically, the learning classifier f=h∘ϕ comprises a feature extractor ϕ and a classifier head *h*. Prior research in UniDA primarily focuses on fine-tuning ϕ using ImageNet pre-trained backbones. In our study, we aim to explore the training of *f* and the scoring function *s* using foundation models such as CLIP backbones.

## 4. Proposed Method

Given a classifier $f:R^{d}\to R^{|Y^{s}|}$ before softmax layer, the output probability of each target instance $x^{t}$ is:

(1) $$p\left(x^{t}\right)=\sigma \left(f\left(x^{t}\right)/\tau \right),$$

where σ is the softmax function and τ is the scaling temperature. In UniDA, since we do not have a validation set and lack prior knowledge about target categories, we do not treat τ as a fixed hyperparameter but aim to automatically learn such a temperature τ for each specific UniDA task.

### 4.1. Motivations

**Figure 1:**
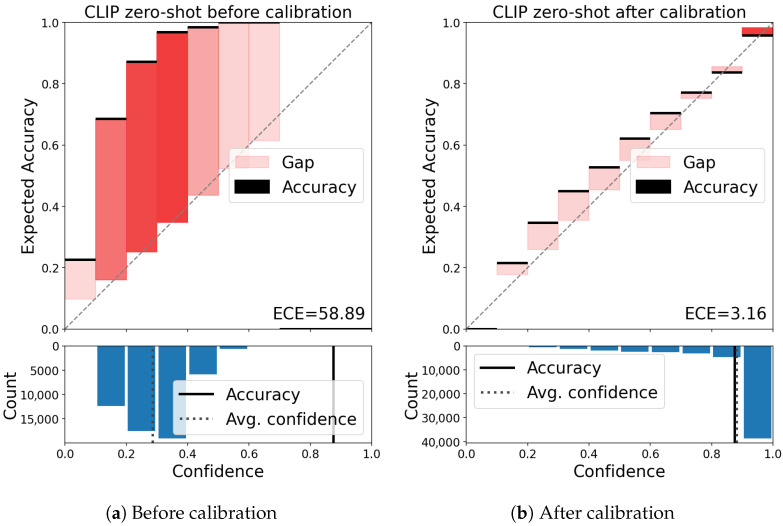
Reliability diagrams (**top**) and confidence histograms (**bottom**) for CLIP zero-shot model before and after calibration on VisDA dataset. (best viewed in color).

**Why scaling the logits?** In UniDA tasks, however, the objective is not solely closed-set classification but also demands effective out-of-class detection. We argue that a well-calibrated model plays a pivotal role in achieving this goal. To gain a clearer perspective on our argument, we illustrate two reliability diagrams [45] comparing the CLIP zero-shot model before and after calibration in Figure 1. As depicted in the figure, without logit scaling, the CLIP zero-shot method tends to classify most samples with low confidence, even if they are classified correctly. This would readily lead to misidentifying the majority of in-of-class samples as out-of-class ones, causing a decrease in in-of-class classification performance. After calibration through temperature scaling, significantly improved confidence estimates can be observed, resulting in a more trustworthy prediction system. Therefore, we scale the logits to ensure the model’s proper calibration and enhance its performance in both out-of-class detection and in-of-class discrimination [46].

### 4.2. Learning Temperature Scaling by Source Confidence Calibration

Different from previous calibration methods [46,47,48] that were proposed for closed-set classification, confidence calibration by temperature scaling faces a challenge for UniDA tasks since we do not have prior knowledge about the target categories. To address this challenge, we propose to learn using the source data. We evenly divide the source data into two parts by class. The first part of the samples is treated as in-of-class samples for IID (In-Identical-Distribution) calibration, while the second part of the samples is treated as out-of-class samples for OOD calibration.

**IID calibration.** Given a ground truth joint distribution $\pi_{in}\left(X,Y\right)=\pi_{in}\left(Y|X\right)\pi_{in}\left(X\right)$, the expected calibration error (ECE) for a prediction model is defined as

(2) $$E_{\hat{P}}[|P\left(\hat{Y}=Y|\hat{P}=p\right)-p\left|\right],$$

where $\hat{Y}$ is a class prediction and $\hat{P}$ is its associated confidence, i.e., probability of correctness. ECE could be approximated by partitioning predictions into *K* equally-spaced bins (similar to the reliability diagrams, see Figure 1) and taking a weight average of the bins’ accuracy/confidence difference [47]. Specifically,

(3) $${\mathrm{ECE}}_{in}=\sum_{k=1}^{K}\frac{|B_{k}|}{n_{in}}\left|\mathrm{acc}\left(B_{k}\right)-\mathrm{conf}\left(B_{k}\right)\right|,$$

where $n_{in}$ is the number of in-of-class samples.

**OOD calibration.** For out-of-class samples ${\left\{x_{i}\right\}}_{i=1}^{n_{out}}$, which do not belong to any specific predicted category, our objective is to maintain a uniform distribution of their output class probabilities:

(4) $${\mathrm{ECE}}_{out}=\frac{1}{n_{out}}\sum_{i=1}^{n_{out}}\left|\mathrm{conf}\left(x_{i}\right)-\frac{1}{N}\right|,$$

where *N* is the number of in-of-class categories.

**Negative log likelihood.** As a standard measure of probabilistic model’s quality [49], negative log likelihood (NLL) is widely used in the context of deep learning, which is also known as the cross entropy loss. Given the known ground truth of in-of-class samples and a prediction probabilistic model ${\hat{\pi }}_{in}\left(Y|X\right)$, NLL is defined as:

(5) $${\mathrm{NLL}}_{in}=-\sum_{i=1}^{n_{in}}\log\left({\hat{\pi }}_{in}\left(y_{i}|x_{i}\right)\right)$$

${\mathrm{NLL}}_{in}$ is minimized if and only if ${\hat{\pi }}_{in}\left(Y|X\right)$ recovers the ground truth conditional distribution $\pi_{in}\left(Y|X\right)$.

Our overall objective of learning temperature scaling is then written as

(6) $$\tau_{opt}=\arg\min_{\tau }{\mathrm{ECE}}_{in}+{\mathrm{ECE}}_{out}+{\mathrm{NLL}}_{in}.$$

## 5. Evaluation and Discussion

In the context of UniDA, the dual objective includes the task of accurately classifying samples from the correct classes $Y^{st}$ while simultaneously rejecting samples from unknown classes $Y^{t/s}$. This dual objective introduces additional complexity to the evaluation of UniDA methods.

### 5.1. Hard Out-of-Class Detection Criteria

The **H-score** metric is introduced with the aim of achieving a balance between the significance of detecting samples outside the class and accurately classifying in-of-class samples, as described in Fu et al.’s work [21]. H-score includes all unknown classes as a superclass and calculates the harmonic mean of the accuracy of the average classes in known classes (acc*_in_*) and the accuracy in the superclass (acc*_out_*), that is, the H-score = 2·acc*_in_*·acc*_out_*/(acc*_in_* + acc*_out_*). While this metric is commonly employed in the UniDA research community, it is important to note that it exhibits a noticeable bias toward the ratios between the quantities of in-of-class and out-of-class samples. Due to the lack of prior knowledge to target data, these ratios may vary in different tasks. It is usually impossible to handle all tasks of different ratios in order to have a fair evaluation between different methods.

It is worth noting that in scenarios where the target data lacks unknown classes, the H-score metric loses its applicability and degenerates into acc*_in_*.

### 5.2. Soft Out-of-Class Detection Criteria

The criterias mentioned above require us to classify a sample as either out-of-class or in-of-class, which means that we have to set a threshold for out-of-class detection. In this paper, we are motivated from the field of open set recognition (Open Set Classification Rate (OSCR) [50] and the Detection and Identification Rate (DIR) [51]) and introduce a new UniDA evaluation metric, which is threshold- and ratio-free. However, unlike the OSR task, which assumes the absence of source private classes and the presence of target private classes, UniDA is more flexible and does not impose such strict constraints. Therefore, we adapt these metrics to accommodate various UniDA scenarios, introducing a new metric called **Universal Classification Rate (UCR)**.

To calculate UCR, we compute a pair of Correct Classification Rate (CCR) and False Positive Rate (FPR) by varying the scoring threshold θ. CCR assesses the proportion of correctly classified in-of-class samples from $D_{in}^{t}$, and FPR quantifies the fraction of out-of-class samples from $D_{out}^{t}$ that are incorrectly detected.

(7) $$\begin{array}{cc} \; & \mathrm{CCR}\left(\theta \right)=\frac{\left|\{x|x\in D_{in}^{t}\wedge f\left(x\right)=\mathrm{label}\left(x\right)\wedge s\left(x\right)>\theta \}\right|}{|D_{in}^{t}|}\\ \; & \mathrm{FPR}\left(\theta \right)=\frac{\left|\{x|x\in D_{out}^{t}\wedge s\left(x\right)>\theta \}\right|}{|D_{out}^{t}|}. \end{array}$$

**Figure 2:**
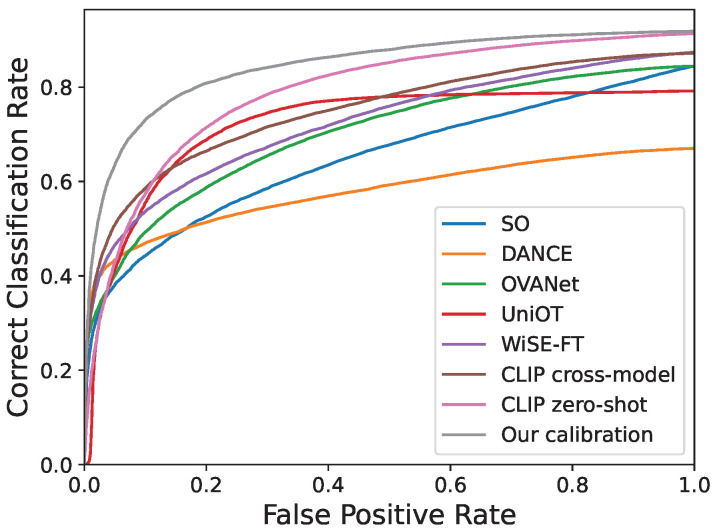
(CCR vs. FPR) curve.

Then, the UCR is calculated as

(8) $$\mathrm{UCR}=\left\{\begin{array}{c} \mathrm{Area}\;\mathrm{Under}\;\mathrm{the}\;(\mathrm{CCR}\;\mathrm{vs}\;\mathrm{FPR})\;\mathrm{Curve},\;\mathrm{if}\;|D_{out}^{t}|>0\\ \mathrm{CCR}(-\infty ),\;\mathrm{if}\;|D_{out}^{t}|=0 \end{array}\right.$$

where, CCR(−∞) is identical to the closed-set classification accuracy on $D_{in}^{t}$. Figure 2 shows an example illustration to the (CCR vs FPR) curve on VisDA task under the (6/3) setting. The distinction between UCR and AUROC lies in the replacement of the true positive rate (TPR) with the correct classification rate (CCR) in UCR. In contrast to previous evaluation metrics, UCR does not rely on thresholds or ratios, making it an additional criterion that does not consider the threshold effects.

## 6. Empirical Results

### 6.1. UniDA Methods Review

We briefly review some representative state-of-the-art (SOTA) methods for comparison: DANCE [22], OVANet [25], and UniOT [26]. In addition to these methods, we consider the Source Only (SO) method as a baseline that involves standard cross-entropy loss training on the source data alone. In inference, the softmax classifier *f* is employed for predictions, and the scoring function *s* is constructed based on the entropy of the softmax output probabilities, following the approach used in DANCE. For a concise overview of these methods, see Table 1.

### 6.2. Datasets and Experimental Setup

**Dataset.** We train the above methods on the standard benchmark datasets for UniDA: Office [52], OfficeHome (OH) [53], VisDA (VD) [54], and DomainNet (DN) [55]. Office has 31 categories and three domains: Amazon (A), DSLR (D), and Webcam (W). OfficeHome contains 65 categories and four domains: Art (A), Clipart (C), Product (P), and Real-World (R) images. VisDA is a synthetic-to-real dataset with 12 categories in total. DomainNet is the largest dataset, including 345 categories and six domains, where three domains—Painting (P), Real (R), and Sketch (S)—are used in experiments following previous work [25,26]. For each dataset, we further split the total categories into three disjoint parts—common categories $Y^{st}$, source private categories $Y^{s/t}$, and target private categories $Y^{t/s}$—to consist of the source and target domains. For a more comprehensive study, we assign each dataset with four different class splits: open-partial, open, closed, partial, following [22]. The different classes splits result in different running tasks, and each split setting, denoted ($|Y^{st}\left|/\right|Y^{s/t}|$), is shown in each table. Detail information about these four datasets and the class-split settings are presented in the Appendix A.

**Implementation.** For fair comparison between different methods, we implement UniOOD, a code framework to streamline rigorous and reproducible experiments in UniDA. By using the UniOOD framework, all methods are run under the same learning setting. For all methods requiring training in our experiments, including the SO baseline and others like DANCE and OVANet, we adopted a consistent linear probing protocol. Specifically, the visual backbone (e.g., CLIP) was kept frozen, and we only trained a linear classifier (a single fully-connected layer followed by softmax) on top of the frozen features. The initial learning rate is set to 0.01 for all new layers and 0.001 for pretrained backbone if it is fine-tuned and decays using the cosine schedule rule with a warm-up of 50 iterations. We use the SGD optimizer with momentum 0.9 and the batch size is set to 32 for each domain. The number of training iterations are set to 5000, 10,000, or 20,000 based on the scale of the training data, which is detailed in Appendix A. We report results of the last checkpoint due to the absence of validation data and average them among three random runs. Due to space constraints, we provide the average results for each split setting, while the detailed results for individual tasks can be found in the Appendix C. The hyperparameters for the previous methods follow their official codes. We do not use any data augmentation during training for fair comparison to different methods, which may be different from previous works.

**Evaluation metrics.** We use H-score and UCR as the evaluation metrics. H-score is a widely used metric for UniDA, and the UCR is introduced in this paper, as detailed in Section 5.

### 6.3. Comparison with SOTA UniDA Methods

Table 2 and Table 3 present the comparative results between our method and existing state-of-the-art (SOTA) UniDA approaches in two distinct evaluation metrics. The results cover four distinct UniDA settings, namely open-partial, open, closed, and partial settings, denoted as different class splits represented as ($|Y^{st}\left|/\right|Y^{s/t}|$) within the table. Regarding the H-score metric, it is evident that our method and the leading state-of-the-art approach are on par with each other in the open-partial and open settings. However, our method demonstrates significant enhancements in all closed and partial settings, achieving improvements that exceed 10% in five out of eight tasks. In terms of the UCR metric, our method significantly outperforms state-of-the-art UniDA methods in three out of four datasets: OfficeHome, VisDA, and DomainNet, across all four settings. In general, our method exhibits superior robustness across various settings and establishes a new state-of-the-art on UniDA benchmarks, excelling in both the H-score metric and the UCR metric.

### 6.4. Comparison with SOTA CLIP-Adaptation Methods

Recall that our focus is on developing a UniDA method based on foundation models like CLIP. Therefore, we also provide comparisons with some state-of-the-art (SOTA) adaptation methods that leverage CLIP models, even though they were not originally designed for the UniDA task. These methods include CLIP zero-shot (baseline) [1], WiSE-FT [7], and CLIP cross-model [5]. WiSE-FT is a new fine-tuning method to improve robustness by ensembling the weights of the zero-shot and fine-tuned models. CLIP cross-model is a recent study introduced by Lin et al. [5], which has demonstrated the most remarkable few-shot capability to date by leveraging cross-model information. However, as all these methods can not directly be used for UniDA, we construct a scoring function *s* following the SO method except for CLIP zero-shot, as illustrated in Table 1. While these methods have shown remarkable enhancements in closed-set robustness benchmarks such as ImageNet, they frequently exhibit lower performance than the SOTA UniDA methods when evaluated using the H-score metric on UniDA benchmarks, as shown in Table 2. However, it should be noted that all these adaptation methods consistently outperform the SOTA UniDA methods when considering the UCR metric, as illustrated in Table 3. Our method maintains its position as the most powerful performer in terms of both H-score and UCR evaluation metrics.

The CLIP calibration method [48] that was proposed for closed-set classification, is exactly equal to our IID calibration when using source data as the auxiliary dataset, which is a component of our overall calibration. The results in Table 2 show that while CLIP calibration [48] slightly outperforms our calibration in closed-set/partial scenarios, it performs significantly worse in open-partial/open-set scenarios. Since we do not have prior knowledge about which scenario to consider, our calibration method is more robust and suitable for UniDA tasks.

### 6.5. Analysis and Ablation Study

**Temperature scaling is necessary.** To demonstrate the effectiveness of our temperature scaling, we report the results of the CLIP model when setting τ=1, as shown in Table 2. It is apparent that without temperature scaling, CLIP model fails to distinguish samples between in-of-class and out-of-class categories, leading to nearly zero performance on the H-score metric. This demonstrates the necessity of temperature scaling and the superiority of our self-calibration method.

**Each calibration loss plays a key role.** We conducted ablation studies to assess the significance of each calibration loss component in our method, namely ECE*_in_*, ECE*_out_*, and NLL*_in_*, corresponding to IID calibration, OOD calibration, and NLL calibration, respectively. The results of this analysis are presented in Table 4. It is evident that the absence of either IID or NLL calibration leads to a substantial decrease in performance in the closed and partial settings. Conversely, the lack of OOD calibration affects the results in the open-partial and open settings. In summary, each calibration loss contributes significantly to the calibration process.

**Simply setting a fixed temperature scaling is not optimal.** To validate the effectiveness of the proposed self-calibration method, we present the results of our approach with fixed temperature scaling using various values, as depicted in Table 5. While the UCR remains consistently stable, the H-score is noticeably impacted by different temperature scaling settings. It is evident that maintaining a fixed temperature scaling is not the optimal solution due to its sensitivity to diverse tasks. Across all tasks and settings, our self-calibration method with automatic temperature scaling proves to be adaptive, showcasing its efficacy. Furthermore, our method, without requiring target validation, could be applied more practically.

## 7. Conclusions, Limitations and Future Work

In this paper, we establish a new, simple, and powerful baseline for UniDA using foundation models. Through comprehensive experiments, we first demonstrate that existing state-of-the-art UniDA methods face significant limitations when applied to foundation models like CLIP. Our key finding reveals that a properly calibrated CLIP model alone consistently outperforms these complex methods across various UniDA scenarios. This leads to our main contribution: a straightforward confidence calibration approach that sets a new state-of-the-art for UniDA with foundation models. The dramatic performance improvements achieved by our minimalistic method underscore that previous methodological complexities may be unnecessary when leveraging foundation models. We unequivocally position our calibrated CLIP as the new strong baseline for future UniDA research, hoping to steer the field toward more efficient and effective use of foundation models rather than increasingly intricate algorithms.

A limitation of this work is that we primarily explore the use of frozen foundation models, as our initial experiments indicated that full fine-tuning of these models led to subpar performance (see Appendix B). Our study thus focuses on leveraging their robust representations as-is, rather than adapting them through extensive retraining. However, recent studies have introduced new techniques for improving fine-tuning with these models, such as the fine-tuning pre-trained methods [56] and surgical fine-tuning [8]. We did not explore these techniques due to the substantial computational resources required, leaving this as a potential avenue for future research.

## Figures and Tables

**Table 1:** A brief introduction of different methods. Previous approaches are categorized into three groups: SO, the baseline method that trains models solely on source data; DANCE [22], OVANet [25], and UniOT [26], state-of-the-art methods designed explicitly for the UniDA task; and WiSE-FT [7], CLIP cross-model [5], and CLIP zero-shot [1], three SOTA methods for CLIP model adaptation. In this paper, we introduce UniDA calibration, which is different from previous calibration method [48] that is designed for closed-set classification, as detailed in Section 4.

Method Type	Methods	Source	Target	Classifier	Scoring Rule	Threshold Value
Baseline	Source Only (SO)	**✓**	**✗**	softmax	negative entropy	$-\log(|Y^{s}|)/2$
UniDA SOTAs	DANCE [22]	**✓**	**✓**	softmax	negative entropy	$-\log(|Y^{s}|)/2$
	OVANet [25]	**✓**	**✓**	softmax	binary softmax prob.	1/2
	UniOT [26]	**✓**	**✓**	OT	maximum OT mass	$1/(n^{t}+T)$
CLIP adaptations	WiSE-FT [7]	**✓**	**✗**	softmax	negative entropy	$-\log(|Y^{s}|)/2$
	CLIP cross-model [5]	**✓**	**✗**	softmax	negative entropy	$-\log(|Y^{s}|)/2$
	CLIP zero-shot [1]	**✗**	**✗**	NN	maximum logit	-
Calibration	CLIP calibration [48]	**✓** *	**✗**	softmax	negative entropy	$-\log(|Y^{s}|)/2$
	Our calibration	**✓** *	**✗**	softmax	negative entropy	$-\log(|Y^{s}|)/2$

* Note that these methods use the source data for confidence calibration, not for training. The symbols **✓** and **✗** denote the utilization and non-utilization, respectively, of corresponding domain data for model training. “prob.” is an abbreviation for probability.

**Table 2:** Comparison results of **H-score** between existing methods and the proposed method using the same CLIP backbone (ViT-L) in four UniDA settings (open-partial, open, closed, and partial scenarios respectively). The best results are highlighted in **bold**, while the second best results are highlighted in blue, and the data indicating significantly poor results (<≈10% to the best) are marked in red.

Methods	Office				OfficeHome				VisDA				DomainNet				Avg
	(10/10)	(10/0)	(31/0)	(10/21)	(10/5)	(15/0)	(65/0)	(25/40)	(6/3)	(6/0)	(12/0)	(6/6)	(150/50)	(150/0)	(345/0)	(150/195)	
SO	91.98	91.87	80.22	89.79	84.52	82.05	58.12	58.31	69.85	75.79	55.31	57.19	61.49	65.63	38.27	35.88	68.52
DANCE [22]	**94.7**	96.09	75.76	66.83	89.01	83.95	55.42	46.63	71.9	74.3	58.08	49.5	60.53	65.24	37.56	30.92	66.03
OVANet [25]	93.36	91.16	74.64	87.53	85.42	80.29	64.65	65.92	59.47	39.27	43.55	42.58	70.7	72.4	57.22	55.86	67.75
UniOT [26]	92.32	**96.48**	59.95	41.31	**89.45**	**86.64**	59.27	43.6	79.1	**83.08**	71.62	62.03	71.42	73.21	63.72	55.18	70.52
WiSE-FT [7]	82.34	94.07	47.87	53.57	79.37	73.44	13.64	16.56	62.68	72.21	30.05	27.4	3.74	7.92	0.3	0.29	41.59
CLIP cross-model [5]	93.04	93.59	83.21	92.55	86.2	84.26	62.65	63.14	77.69	81.66	62.08	67.98	61.98	67.1	36.2	34.06	71.71
w/o calibration (τ=1)	0.0	0.07	0.0	0.0	0.17	1.31	0.0	0.0	0.0	0.05	0.0	0.0	0.0	0.0	0.0	0.0	0.1
CLIP calibration [48]	35.18	38.72	**87.53**	**96.84**	47.02	46.9	**87.01**	**87.1**	74.49	46.53	**82.2**	**87.87**	58.57	58.78	**77.57**	**78.28**	68.16
Our calibration	86.74	91.89	83.8	94.39	86.4	84.77	80.58	80.81	**84.74**	82.26	76.08	83.12	**72.37**	**74.22**	74.93	75.22	**82.02**

**Table 3:** Comparison results of **UCR** between existing methods and the proposed method using the same CLIP backbone (ViT-L) in four UniDA settings (open-partial, open, closed, and partial scenarios respectively). The best results are highlighted in **bold**, while the second best results are highlighted in blue, and the data indicating significantly poor results (<≈10% to the best) are marked in red.

Methods	Office				OfficeHome				VisDA				DomainNet				Avg
	(10/10)	(10/0)	(31/0)	(10/21)	(10/5)	(15/0)	(65/0)	(25/40)	(6/3)	(6/0)	(12/0)	(6/6)	(150/50)	(150/0)	(345/0)	(150/195)	
SO	93.98	94.95	91.44	96.99	86.89	84.53	83.55	84.44	63.46	71.17	76.66	79.51	63.19	66.01	71.26	70.78	79.93
DANCE [22]	95.17	97.09	87.69	81.1	90.33	86.76	81.74	75.63	57.78	63.45	67.86	56.4	64.88	68.37	71.66	67.6	75.84
OVANet [25]	95.36	95.8	91.4	96.84	88.18	85.72	83.52	84.37	68.57	66.45	76.68	79.58	64.3	66.94	71.37	70.94	80.38
UniOT [26]	90.62	97.2	92.14	55.94	88.85	85.59	85.5	63.61	72.22	78.8	84.79	74.78	62.88	67.33	73.85	67.99	77.63
WiSE-FT [7]	95.27	96.33	92.3	97.57	90.77	89.28	87.28	88.44	70.83	77.88	81.45	84.43	68.72	71.66	75.74	75.77	83.98
CLIP cross-model [5]	**95.38**	96.18	**93.24**	**97.58**	89.71	87.82	86.95	87.97	73.22	79.06	81.15	83.76	68.81	71.53	75.57	75.55	83.97
CLIP zero-shot [1]	90.1	97.68	87.69	96.61	90.21	89.67	89.08	89.43	78.6	82.86	87.56	88.1	70.78	73.34	79.48	79.87	85.69
w/o calibration (τ=1)	92.46	97.75	87.68	96.61	92.91	91.71	89.08	89.41	80.9	85.75	87.56	88.1	69.2	72.81	79.48	**79.88**	86.33
CLIP calibration [48]	90.88	97.55	87.69	96.61	92.24	90.59	89.08	89.43	**82.8**	86.32	87.56	88.1	74.57	76.88	79.49	79.87	86.85
Our calibration	93.39	**97.91**	87.69	96.61	**93.02**	**91.73**	**89.08**	**89.43**	82.38	**86.37**	**87.56**	**88.1**	**74.86**	**77.21**	**79.49**	79.87	**87.17**

**Table 4:** Ablation studies. The best results are highlighted in **bold**, while data indicating significantly poor results (<≈10% to the best) are marked in red. It exhibits enhanced stability and robustness across diverse task settings when incorporating all calibration losses.

Methods	Office				OfficeHome				VisDA				DomainNet				Avg
	(10/10)	(10/0)	(31/0)	(10/21)	(10/5)	(15/0)	(65/0)	(25/40)	(6/3)	(6/0)	(12/0)	(6/6)	(150/50)	(150/0)	(345/0)	(150/195)	
H-score																	
Only IID calibration	35.18	38.72	**87.53**	**96.84**	47.02	46.9	87.01	87.1	74.49	46.53	82.2	87.87	58.57	58.78	77.57	78.28	68.16
Only NLL calibration	36.7	66.24	87.01	96.48	53.41	53.27	87.33	87.38	72.45	45.81	**85.52**	**89.84**	58.24	59.21	77.59	**78.3**	70.92
Only OOD calibration	0.0	0.0	0.0	0.0	0.0	0.0	0.0	0.0	0.0	0.0	0.0	0.0	0.0	0.0	0.0	0.0	0.0
w/o IID calibration	**90.11**	76.44	77.8	89.3	**87.61**	84.68	72.46	73.4	82.33	58.11	58.03	62.25	77.38	79.01	70.63	70.17	75.61
w/o NLL calibration	88.63	73.38	75.33	86.84	87.39	84.03	66.58	68.01	78.85	54.6	54.96	58.32	**77.38**	**79.14**	67.66	66.66	72.98
w/o OOD calibration	35.36	55.43	87.01	96.48	56.4	56.23	**87.37**	**87.42**	72.69	45.53	82.3	87.99	59.59	58.65	**77.59**	78.28	70.27
Ours	86.74	**91.89**	83.8	94.39	86.4	**84.77**	80.58	80.81	**84.74**	**82.26**	76.08	83.12	72.37	74.22	74.93	75.22	**82.02**
UCR																	
Only IID calibration	90.88	97.55	87.69	96.61	92.24	90.59	89.08	89.43	82.8	86.32	87.56	88.1	74.57	76.88	79.49	79.87	86.85
Only NLL calibration	90.52	97.7	87.69	96.61	92.39	90.78	89.08	89.43	82.83	86.31	87.56	88.1	74.57	76.89	79.49	79.87	86.86
Only OOD calibration	86.98	96.86	87.69	96.61	91.69	90.49	89.08	89.43	78.91	84.55	87.56	88.1	57.83	62.48	79.49	79.87	84.23
w/o IID calibration	93.51	97.88	87.69	96.61	93.07	91.84	89.08	89.43	82.2	86.16	87.56	88.1	**74.92**	**77.26**	79.49	79.87	87.17
w/o NLL calibration	**93.51**	97.88	87.69	96.61	**93.08**	**91.85**	89.08	89.43	82.1	86.15	87.56	88.1	74.9	77.25	79.49	79.87	87.16
w/o OOD calibration	90.38	97.65	87.69	96.61	92.45	90.87	89.08	89.43	**82.83**	86.31	87.56	88.1	74.6	76.87	79.49	79.87	86.86
Ours	93.39	**97.91**	**87.69**	**96.61**	93.02	91.73	**89.08**	**89.43**	82.38	**86.37**	**87.56**	**88.1**	74.86	77.21	**79.49**	**79.87**	**87.17**

**Table 5:** Comparison to that of fixed temperature scaling with various values. The best results are highlighted in **bold**, while data indicating significantly poor results (<≈10% to the best) are marked in red. Setting different values of τ has little impact to the UCR, but it significantly affects the H-score. The results illustrate that fixing a scaling value is sensitive to various tasks, while our method is robust across diverse settings. This demonstrates the efficacy of the proposed self-calibration method. Given the absence of a validation set in UniDA, our method offers a robust solution for adapting to diverse tasks.

Methods	Office				OfficeHome				VisDA				DomainNet				Avg
	(10/10)	(10/0)	(31/0)	(10/21)	(10/5)	(15/0)	(65/0)	(25/40)	(6/3)	(6/0)	(12/0)	(6/6)	(150/50)	(150/0)	(345/0)	(150/195)	
H-score																	
τ= 0.1	17.79	27.94	**87.53**	**96.84**	20.31	20.3	**88.35**	**88.41**	32.13	41.24	**89.6**	**92.06**	4.55	6.13	**79.34**	**80.1**	54.54
τ= 0.2	62.28	71.5	87.27	96.69	60.49	60.33	86.75	86.9	67.96	71.96	84.37	89.21	62.49	63.83	77.47	78.17	75.48
τ= 0.3	83.28	90.45	84.7	95.16	81.09	80.49	80.06	80.24	83.41	86.6	75.01	82.37	**77.54**	**79.58**	63.02	61.75	80.3
τ= 0.4	**90.45**	**95.51**	75.13	88.0	**88.95**	**87.09**	62.12	64.09	83.03	**87.32**	62.15	67.33	56.51	63.38	26.64	26.75	70.28
τ= 0.5	80.04	93.82	50.79	55.82	88.18	84.77	32.85	38.97	68.3	80.4	39.98	40.39	15.82	24.23	2.61	3.04	50.0
τ= 0.6	59.36	81.49	24.95	25.91	78.42	73.0	7.9	12.97	39.74	63.18	14.31	12.63	1.61	3.1	0.1	0.14	31.18
τ= 0.7	33.69	64.05	6.76	3.35	57.83	50.18	1.07	2.26	10.95	34.87	3.2	1.47	0.05	0.16	0.0	0.01	16.87
τ= 0.8	2.07	41.75	0.38	0.0	26.6	22.82	0.13	0.31	1.15	8.61	0.07	0.06	0.01	0.01	0.0	0.0	6.5
τ= 0.9	0.0	8.68	0.03	0.0	4.64	5.09	0.0	0.02	0.0	1.38	0.0	0.0	0.0	0.0	0.0	0.0	1.24
τ= 1.0	0.0	0.07	0.0	0.0	0.17	1.31	0.0	0.0	0.0	0.05	0.0	0.0	0.0	0.0	0.0	0.0	0.1
Ours	86.74	91.89	83.8	94.39	86.4	84.77	80.58	80.81	**84.74**	82.26	76.08	83.12	72.37	74.22	74.93	75.22	**82.02**
UCR																	
τ= 0.1	90.14	97.49	87.69	96.61	91.72	89.85	89.08	89.43	**82.98**	86.23	87.56	88.1	72.3	74.72	79.49	79.87	86.45
τ= 0.2	92.22	97.73	87.69	96.61	92.5	90.94	89.08	89.43	82.89	86.61	87.56	88.1	74.67	77.0	79.49	79.87	87.02
τ= 0.3	93.26	97.86	87.69	96.61	92.92	91.56	89.08	89.43	82.57	**86.63**	87.56	88.1	74.86	**77.23**	79.49	79.87	87.17
τ= 0.4	**93.52**	97.9	87.69	96.61	93.06	91.8	89.08	89.43	82.23	86.5	87.56	88.1	74.4	76.89	79.49	79.87	87.13
τ= 0.5	93.47	97.91	87.69	96.61	**93.1**	91.88	89.08	89.43	81.93	86.34	87.56	88.1	73.7	76.35	79.49	79.87	87.03
τ= 0.6	93.31	97.89	87.69	96.61	93.08	**91.88**	89.08	89.43	81.66	86.19	87.56	88.1	72.87	75.7	79.49	79.87	86.9
τ= 0.7	93.09	97.86	87.69	96.61	93.05	91.86	89.08	89.43	81.43	86.06	87.56	88.1	71.96	74.99	79.49	79.87	86.76
τ= 0.8	92.91	97.83	87.69	96.61	93.0	91.82	89.08	89.43	81.23	85.94	87.56	88.1	71.03	74.26	79.49	79.87	86.62
τ= 0.9	92.69	97.79	87.69	96.61	92.96	91.77	89.08	89.43	81.06	85.84	87.56	88.1	70.11	73.53	79.49	79.87	86.47
τ= 1.0	92.46	97.75	87.69	96.61	92.91	91.71	89.08	89.43	80.9	85.75	87.56	88.1	69.2	72.81	79.49	79.87	86.33
Ours	93.39	**97.91**	**87.69**	**96.61**	93.02	91.73	**89.08**	**89.43**	82.38	86.37	**87.56**	**88.1**	**74.86**	77.21	**79.49**	**79.87**	**87.17**

## Data Availability

The data supporting this study are publicly available.

## Abbreviations

The following abbreviations are used in this manuscript:

DA	Domain Adaptation
UniDA	Universal Domain Adaptation
IID	In Identical Distribution
OOD	Out Of Distribution
OSR	Open-Set Recognition
UCR	Universal Classification Rate
SO	Source Only
NLL	Negative Log Likelihood
SOTA	State-Of-The-Art

## Appendix A. Experimental Setup Details

### Appendix A.1. Dataset

We provide detail information about four datasets—Office [52], OfficeHome (OH) [53], VisDA (VD) [54], and DomainNet (DN) [55]—in Table A1.

**Table A1:** Datasets information.

Office (31 categories)	Domains	Amazon (A)	DSLR (D)	Webcam (W)	-
	Number of Samples	2817	498	795	-
OfficeHome (65 categories)	Domains	Art (A)	Clipart (C)	Product (P)	RealWorld (R)
	Number of Samples	2427	4365	4439	4357
VisDA (12 categories)	Domains	Syn (S)	Real (R)	-	-
	Number of Samples	152,397	55,388	-	-
DomainNet (345 categories)	Domains	Painting (P)	Real (R)	Sketch (S)	-
	Number of Samples	50,416	120,906	48,212	-

### Appendix A.2. Classes Split Settings

The total categories of each dataset are split into the three disjoint parts—common categories $Y^{st}$, source private categories $Y^{s/t}$, and target private categories $Y^{t/s}$—to consist source and target domains. Since $|Y^{st}\left|+\right|Y^{s/t}\left|+\right|Y^{t/s}|$ is fixed, we name each split setting as ($|Y^{st}\left|/\right|Y^{s/t}|$). The split settings for different datasets are shown in Table A2, following previous setting protocols [22].

**Table A2:** Classes split settings on four datasets.

Datasets	Split Settings			
	Open-Partial	Open	Closed	Partial
Office	(10/10)	(10/0)	(31/0)	(10/21)
OfficeHome	(10/5)	(15/0)	(65/0)	(25/40)
VisDA	(6/3)	(6/0)	(12/0)	(6/6)
DomainNet	(150/50)	(150/0)	(345/0)	(150/195)

### Appendix A.3. Number of Training Iterations

The maximum number of training iterations for the model is determined based on the scale of the training dataset. It is set to either 5000, 10,000, or 20,000 for different task settings, as indicated in Table A3.

**Table A3:** Training iterations on different task settings.

Datasets	Split Settings			
	Open-Partial	Open	Closed	Partial
Office	5000	5000	10,000	10,000
OfficeHome	5000	5000	10,000	10,000
VisDA	10,000	10,000	20,000	20,000
DomainNet	10,000	10,000	20,000	20,000

### Appendix A.4. Other Settings

**Text template using for CLIP zero-shot method**: We follow the ensemble text templates in [5] for CLIP zero-shot method, which include 180 templates. Each class prototype is calculated as the mean vector of the 180 corresponding text encoding vectors.

**Compute description**: Our computing resource is a single GPU of NVIDIA GeForce RTX 3090 with 32 Intel(R) Xeon(R) Silver 4215R CPU @ 3.20 GHz.

**Existing codes used**: To fair comparison to different methods, we build a code framework—UniOOD, which integrates many previous methods. All codes to implement previous methods are directly copied from their official codes:

DANCE [22]: https://github.com/VisionLearningGroup/DANCE, accessed on 27 November 2025.

OVANet [25]: https://github.com/VisionLearningGroup/OVANet, accessed on 27 November 2025.

UniOT [26]: https://github.com/changwxx/UniOT-for-UniDA, accessed on 27 November 2025.

WiSE-FT [7]: https://github.com/mlfoundations/wise-ft, accessed on 27 November 2025.

CLIP cross-model [5]: https://github.com/linzhiqiu/cross_modal_adaptation, accessed on 27 November 2025.

The use of CLIP models [1] follows https://github.com/openai/CLIP, accessed on 27 November 2025.

**Additional evaluation metric: H^3^-score**: Additionally, the quality of clustering for target private samples is introduced as an auxiliary objective in [26] to aid in identifying target private classes. The H^3^-score is calculated as 3/((1/acc*_in_*) + (1/acc*_out_*) + (1/NMI)), where Normalized Mutual Information (NMI) [57] is the widely used metric for clustering.

## Appendix B. Justification for Frozen Foundation Model Backbone

As shown in Table A4, our comparison of various backbones reveals a critical trend specific to foundation models: while full fine-tuning remains effective for the standard ImageNet-pretrained model, it consistently degrades performance for foundation models like DINOv2 and CLIP, yielding results that offer little to no gain over a from-scratch baseline. Conversely, when these foundation models are kept frozen, they enable UniDA methods (including the Source-Only baseline) to achieve substantially stronger performance, with CLIP providing the most significant gains. This clear empirical evidence establishes that using a frozen backbone is the superior and necessary strategy for leveraging foundation models in UniDA, which is consequently adopted as a core protocol in our work. Code to reproduce all experiments is available at: https://github.com/szubing/uniood, accessed on 27 November 2025.

**Table A4:** Comparison results using ViT-B [58] backbone with various pre-trained models and fine-tuning modes. Results are conducted on the VisDA dataset in the open-partial UniDA setting.

Methods	ImageNet-Pretrained			Random Initialization			DINOv2-Pretrained			CLIP-Pretrained		
	H-Score	H^3^-Score	UCR	H-Score	H^3^-Score	UCR	H-Score	H^3^-Score	UCR	H-Score	H^3^-Score	UCR
Full Fine-tuning backbone												
SO	58.16	63.73	52.31	8.01	1.45	6.78	0.54	0.46	7.16	2.15	1.95	8.46
DANCE [22]	42.36	49.17	28.36	0.82	1.02	5.81	0.42	0.44	5.94	0.46	0.58	5.98
OVANet [25]	38.27	45.60	53.63	1.55	1.23	7.36	6.65	0.82	5.51	1.06	1.08	4.40
UniOT [26]	71.23	70.93	65.52	11.95	2.17	9.44	7.56	2.13	5.98	15.02	2.67	8.42
Freeze backbone												
SO	56.69	62.19	52.90	14.16	1.73	5.09	57.63	65.16	53.12	70.30	73.58	67.28
DANCE [22]	42.59	50.07	28.12	10.08	1.67	6.40	44.79	53.58	34.53	67.79	71.73	54.17
OVANet [25]	56.36	62.75	67.77	6.55	1.58	4.30	57.91	65.40	48.86	56.36	62.75	67.77
UniOT [26]	68.26	67.16	62.25	11.24	1.39	7.59	62.73	67.12	52.27	75.87	74.81	67.58

## Appendix C. Detail Experimental Results

**Table A5:** Office: ResNet50 & (10/10) setting. Best results are highlighted in bold.

Methods	A2D	A2W	D2A	D2W	W2A	W2D	Avg
H-score							
SO	54.48 ± 3.1	53.47 ± 1.48	79.11 ± 0.25	76.59 ± 1.0	73.56 ± 1.42	60.82 ± 1.84	66.34
DANCE	77.87 ± 0.94	75.97 ± 0.92	77.0 ± 1.74	90.92 ± 2.59	72.37 ± 2.69	87.79 ± 2.16	80.32
OVANet	**82.95** ± 0.56	**77.61** ± 1.19	67.91 ± 2.24	**94.99** ± 0.3	81.25 ± 0.37	**95.26** ± 0.27	83.33
UniOT	77.09 ± 1.11	76.73 ± 0.57	**86.44** ± 0.42	91.22 ± 0.79	**85.45** ± 0.53	89.3 ± 0.52	**84.37**
H^3^-score							
SO	60.18 ± 2.76	59.35 ± 1.0	66.71 ± 0.44	75.07 ± 0.61	65.44 ± 0.49	66.54 ± 1.46	65.55
DANCE	73.08 ± 1.55	70.89 ± 0.66	64.03 ± 1.56	79.95 ± 1.89	62.11 ± 1.63	79.35 ± 1.81	71.57
OVANet	**80.45** ± 0.67	**76.76** ± 1.04	59.72 ± 1.45	**87.29** ± 1.24	67.26 ± 0.37	**89.29** ± 0.93	76.8
UniOT	76.88 ± 2.23	75.02 ± 0.64	**69.41** ± 1.86	86.17 ± 1.65	**68.86** ± 1.4	87.66 ± 0.74	**77.33**
UCR							
SO	69.72 ± 1.45	62.83 ± 2.31	79.54 ± 0.62	94.19 ± 1.27	82.78 ± 0.9	98.2 ± 0.22	81.21
DANCE	**79.04** ± 2.5	**79.86** ± 0.87	82.61 ± 0.48	93.21 ± 1.29	81.68 ± 0.72	90.42 ± 2.18	84.47
OVANet	71.79 ± 0.64	65.18 ± 1.03	73.98 ± 2.03	**97.3** ± 0.67	81.46 ± 0.44	**98.54** ± 0.14	81.38
UniOT	72.66 ± 2.24	72.81 ± 2.18	**87.12** ± 0.98	94.74 ± 0.75	**87.51** ± 0.5	93.57 ± 2.33	**84.73**

**Table A6:** Office: CLIP & (10/10) setting. Best results are highlighted in bold.

Methods	A2D	A2W	D2A	D2W	W2A	W2D	Avg
H-score							
SO	92.71 ± 0.13	89.3 ± 0.19	90.08 ± 0.21	93.95 ± 0.17	88.16 ± 0.22	97.69 ± 0.2	91.98
DANCE	**96.02** ± 0.2	90.18 ± 0.83	93.68 ± 2.56	**98.64** ± 0.21	90.23 ± 2.67	**99.42** ± 0.24	**94.69**
OVANet	93.82 ± 0.58	88.88 ± 0.63	92.3 ± 2.09	97.63 ± 0.0	89.35 ± 0.21	98.16 ± 0.0	93.36
UniOT	84.59 ± 1.64	**92.24** ± 1.23	**94.5** ± 1.58	94.84 ± 1.79	**94.76** ± 0.95	92.99 ± 0.61	92.32
WiSE-FT	77.89 ± 0.42	70.19 ± 0.1	80.83 ± 0.22	92.68 ± 0.17	76.75 ± 0.33	95.7 ± 0.37	82.34
CLIP cross-model	94.24 ± 0.39	89.82 ± 0.32	92.34 ± 0.11	93.65 ± 0.3	92.23 ± 0.05	95.99 ± 0.24	93.05
Our calibration	91.47 ± 0.0	85.13 ± 0.0	85.3 ± 0.0	82.04 ± 0.0	86.46 ± 0.0	90.06 ± 0.0	86.74
H^3^-score							
SO	92.41 ± 0.09	91.54 ± 0.13	83.22 ± 0.12	94.74 ± 0.12	82.12 ± 0.13	95.65 ± 0.13	89.95
DANCE	**94.58** ± 0.13	**92.15** ± 0.58	**85.22** ± 1.4	**97.87** ± 0.14	83.29 ± 1.53	**96.75** ± 0.15	**91.64**
OVANet	93.14 ± 0.38	91.24 ± 0.44	84.47 ± 1.17	97.2 ± 0.0	82.81 ± 0.12	95.95 ± 0.0	90.8
UniOT	87.14 ± 0.7	91.12 ± 0.48	84.31 ± 1.23	94.03 ± 2.03	84.27 ± 1.17	93.56 ± 0.34	89.07
WiSE-FT	82.04 ± 0.31	77.18 ± 0.08	77.74 ± 0.14	93.88 ± 0.11	75.18 ± 0.21	94.37 ± 0.24	83.4
CLIP cross-model	93.41 ± 0.25	91.9 ± 0.22	84.5 ± 0.06	94.54 ± 0.2	**84.43** ± 0.03	94.55 ± 0.16	90.56
Our calibration	91.59 ± 0.0	88.57 ± 0.0	80.45 ± 0.0	86.32 ± 0.0	81.13 ± 0.0	90.64 ± 0.0	86.45
UCR							
SO	88.41 ± 0.08	90.78 ± 0.01	93.09 ± 0.24	98.55 ± 0.09	93.27 ± 0.15	99.8 ± 0.01	93.98
DANCE	**95.96** ± 0.3	**93.03** ± 1.31	92.92 ± 3.15	**99.61** ± 0.03	89.5 ± 2.19	**99.99** ± 0.0	95.17
OVANet	91.39 ± 0.11	92.25 ± 0.45	94.92 ± 0.2	99.18 ± 0.02	94.46 ± 0.26	99.94 ± 0.01	95.36
UniOT	76.31 ± 2.68	90.53 ± 0.4	93.58 ± 1.57	95.05 ± 0.95	94.31 ± 1.24	93.94 ± 0.64	90.62
WiSE-FT	91.82 ± 0.07	92.29 ± 0.05	94.53 ± 0.1	98.52 ± 0.04	95.0 ± 0.08	99.48 ± 0.3	95.27
CLIP cross-model	90.86 ± 0.17	92.18 ± 0.07	**95.49** ± 0.01	98.55 ± 0.01	**95.42** ± 0.01	99.76 ± 0.0	**95.38**
CLIP zero-shot	91.26 ± 0.0	89.87 ± 0.0	89.17 ± 0.0	89.87 ± 0.0	89.17 ± 0.0	91.26 ± 0.0	90.1
Our calibration	93.64 ± 0.0	92.6 ± 0.0	94.0 ± 0.0	92.54 ± 0.0	94.04 ± 0.0	93.54 ± 0.0	93.39

**Table A7:** OfficeHome: ResNet50 & (10/5) setting. Best results are highlighted in bold.

Methods	A2C	A2P	A2R	C2A	C2P	C2R	P2A	P2C	P2R	R2A	R2C	R2P	Avg
H-score													
SO	50.35 ± 0.25	50.87 ± 0.31	55.44 ± 0.59	59.31 ± 1.19	49.15 ± 0.57	57.57 ± 1.09	62.01 ± 0.48	50.21 ± 0.68	56.83 ± 0.41	56.43 ± 0.12	52.31 ± 0.3	52.19 ± 0.41	54.39
DANCE	39.64 ± 3.7	38.23 ± 5.91	38.69 ± 3.66	38.55 ± 3.4	13.22 ± 0.27	37.21 ± 0.43	51.73 ± 2.72	43.89 ± 1.31	43.2 ± 1.78	29.33 ± 4.01	44.47 ± 3.19	50.62 ± 0.88	39.06
OVANet	58.01 ± 0.64	78.91 ± 0.18	82.15 ± 0.56	69.4 ± 0.63	68.1 ± 0.22	76.41 ± 0.08	71.98 ± 0.58	56.77 ± 0.23	81.72 ± 0.11	**77.94** ± 0.55	58.91 ± 0.21	79.81 ± 0.46	71.68
UniOT	**66.13** ± 0.97	**80.42** ± 0.7	**84.56** ± 0.54	**72.79** ± 0.2	**76.59** ± 1.66	**82.42** ± 0.96	**75.82** ± 0.96	**65.87** ± 0.76	**85.07** ± 1.14	76.61 ± 0.3	**64.8** ± 1.23	**80.6** ± 0.44	**75.97**
H^3^-score													
SO	51.29 ± 0.3	57.77 ± 0.29	60.11 ± 0.52	57.45 ± 0.76	55.1 ± 0.45	60.21 ± 0.78	60.58 ± 0.38	50.49 ± 0.28	61.05 ± 0.32	57.72 ± 0.08	52.1 ± 0.12	58.55 ± 0.33	56.87
DANCE	42.95 ± 3.02	45.61 ± 5.5	45.11 ± 3.32	43.56 ± 2.91	18.22 ± 0.34	43.86 ± 0.39	54.24 ± 2.11	46.3 ± 0.88	49.38 ± 1.61	35.1 ± 3.77	46.67 ± 2.44	56.98 ± 0.71	44.0
OVANet	53.85 ± 0.3	77.3 ± 0.23	77.29 ± 0.11	65.06 ± 0.44	68.91 ± 0.25	72.69 ± 0.18	66.75 ± 0.54	53.51 ± 0.29	76.83 ± 0.02	**70.97** ± 0.53	55.14 ± 0.38	77.25 ± 0.29	67.96
UniOT	**59.59** ± 1.0	**78.39** ± 0.47	**78.43** ± 0.36	**65.85** ± 0.31	**74.71** ± 1.07	**75.91** ± 0.74	**69.21** ± 1.03	**58.94** ± 0.6	**79.31** ± 1.22	68.95 ± 0.19	**58.33** ± 0.81	**78.23** ± 0.41	**70.49**
UCR													
SO	38.15 ± 0.75	74.76 ± 0.35	89.28 ± 0.45	61.78 ± 1.34	63.97 ± 0.77	78.44 ± 1.84	62.67 ± 0.39	37.08 ± 0.14	85.66 ± 0.41	69.25 ± 0.91	39.61 ± 0.22	81.54 ± 0.92	65.18
DANCE	**53.53** ± 0.77	72.81 ± 0.82	80.43 ± 1.28	70.95 ± 0.91	69.87 ± 1.65	78.64 ± 1.24	**73.94** ± 0.38	**50.25** ± 0.52	82.14 ± 0.77	69.4 ± 2.15	**52.06** ± 0.82	78.11 ± 0.51	69.34
OVANet	42.81 ± 0.9	77.99 ± 0.1	89.85 ± 0.29	65.89 ± 0.97	65.1 ± 0.36	79.33 ± 0.74	65.7 ± 1.32	39.84 ± 0.03	87.6 ± 0.43	74.79 ± 1.27	42.57 ± 0.35	82.55 ± 0.44	67.83
UniOT	50.72 ± 1.15	**83.24** ± 1.46	**92.86** ± 0.45	**72.98** ± 1.91	**77.33** ± 1.93	**89.54** ± 1.01	68.89 ± 2.22	47.06 ± 1.21	**89.51** ± 0.93	**75.29** ± 1.35	51.6 ± 1.26	**84.75** ± 0.71	**73.65**

**Table A8:** OfficeHome: CLIP & (10/5) setting. Best results are highlighted in bold.

Methods	A2C	A2P	A2R	C2A	C2P	C2R	P2A	P2C	P2R	R2A	R2C	R2P	Avg
H-score													
SO	75.73 ± 0.08	84.45 ± 0.07	88.31 ± 0.02	86.28 ± 0.16	88.14 ± 0.23	91.1 ± 0.12	81.52 ± 0.14	78.42 ± 0.1	89.53 ± 0.13	85.3 ± 0.07	81.68 ± 0.04	83.82 ± 0.09	84.52
DANCE	82.2 ± 0.08	**94.02** ± 0.01	90.2 ± 0.06	86.63 ± 0.16	**93.72** ± 0.16	**93.79** ± 0.02	85.35 ± 0.21	84.47 ± 0.09	91.7 ± 0.11	86.44 ± 0.11	83.97 ± 0.05	**95.64** ± 0.39	89.01
OVANet	80.55 ± 0.4	91.84 ± 0.08	90.74 ± 0.27	86.07 ± 0.17	90.52 ± 0.11	91.78 ± 0.12	75.64 ± 0.67	68.54 ± 0.71	90.29 ± 0.12	85.74 ± 0.2	80.56 ± 0.31	92.75 ± 0.77	85.42
UniOT	**88.28** ± 0.33	92.03 ± 0.56	**93.39** ± 0.31	87.61 ± 0.56	90.19 ± 0.44	92.09 ± 0.82	85.99 ± 0.37	**86.08** ± 0.36	92.04 ± 0.7	86.87 ± 0.13	**86.47** ± 0.43	92.35 ± 0.57	**89.45**
WiSE-FT	68.39 ± 0.04	89.68 ± 0.01	90.9 ± 0.04	69.46 ± 0.27	87.43 ± 0.14	87.14 ± 0.2	68.7 ± 0.45	59.92 ± 0.34	85.8 ± 0.05	78.64 ± 0.26	73.97 ± 0.29	92.43 ± 0.11	79.37
CLIP cross-model	78.37 ± 0.06	85.06 ± 0.05	89.18 ± 0.12	88.98 ± 0.08	88.09 ± 0.24	91.52 ± 0.1	85.91 ± 0.05	82.6 ± 0.11	91.59 ± 0.07	86.8 ± 0.17	82.09 ± 0.05	84.24 ± 0.09	86.2
Our calibration	82.39 ± 0.0	79.09 ± 0.0	85.41 ± 0.0	**89.43** ± 0.0	81.65 ± 0.0	87.21 ± 0.0	**90.06** ± 0.0	84.62 ± 0.0	**93.76** ± 0.0	**90.4** ± 0.0	85.17 ± 0.0	87.64 ± 0.0	86.4
H3-score													
SO	74.95 ± 0.05	86.46 ± 0.05	87.22 ± 0.01	79.88 ± 0.09	89.0 ± 0.16	89.02 ± 0.08	77.1 ± 0.09	76.69 ± 0.06	88.01 ± 0.09	79.31 ± 0.04	78.74 ± 0.02	86.02 ± 0.06	82.7
DANCE	79.06 ± 0.05	**92.91** ± 0.01	88.44 ± 0.04	80.08 ± 0.09	**92.72** ± 0.11	**90.71** ± 0.01	79.34 ± 0.12	80.45 ± 0.06	89.4 ± 0.07	79.97 ± 0.06	80.14 ± 0.03	**93.96** ± 0.25	85.6
OVANet	78.03 ± 0.25	91.48 ± 0.05	88.79 ± 0.17	79.76 ± 0.1	90.61 ± 0.07	89.45 ± 0.08	73.5 ± 0.42	70.1 ± 0.5	88.5 ± 0.07	79.57 ± 0.11	78.04 ± 0.19	92.08 ± 0.51	83.33
UniOT	**83.27** ± 0.32	91.93 ± 0.37	**91.63** ± 0.23	**83.12** ± 0.53	90.97 ± 0.37	90.32 ± 0.58	**82.57** ± 0.81	**82.11** ± 0.1	90.76 ± 0.35	**84.06** ± 0.56	**82.16** ± 0.2	92.22 ± 0.12	**87.09**
WiSE-FT	69.99 ± 0.03	90.04 ± 0.01	88.89 ± 0.02	69.49 ± 0.18	88.52 ± 0.09	86.46 ± 0.13	68.98 ± 0.3	63.84 ± 0.26	85.58 ± 0.03	75.36 ± 0.16	73.79 ± 0.19	91.87 ± 0.07	79.4
CLIP cross-model	76.66 ± 0.04	86.88 ± 0.03	87.79 ± 0.08	81.4 ± 0.05	88.97 ± 0.16	89.29 ± 0.06	79.67 ± 0.03	79.31 ± 0.07	89.33 ± 0.04	80.17 ± 0.09	78.99 ± 0.03	86.31 ± 0.06	83.73
Our calibration	79.18 ± 0.0	82.63 ± 0.0	85.32 ± 0.0	81.65 ± 0.0	84.48 ± 0.0	86.5 ± 0.0	82.0 ± 0.0	80.54 ± 0.0	**90.69** ± 0.0	82.19 ± 0.0	80.87 ± 0.0	88.66 ± 0.0	83.73
UCR													
SO	71.07 ± 0.09	91.59 ± 0.09	95.69 ± 0.06	90.87 ± 0.12	93.28 ± 0.11	95.59 ± 0.06	76.68 ± 0.1	76.23 ± 0.13	94.01 ± 0.04	86.36 ± 0.06	76.35 ± 0.19	95.01 ± 0.08	86.89
DANCE	80.3 ± 0.07	**96.38** ± 0.07	95.85 ± 0.04	90.33 ± 0.07	96.25 ± 0.08	96.87 ± 0.04	80.12 ± 0.12	**85.02** ± 0.17	95.72 ± 0.06	85.97 ± 0.11	82.83 ± 0.12	**98.33** ± 0.03	90.33
OVANet	74.67 ± 0.25	94.7 ± 0.11	96.66 ± 0.08	91.45 ± 0.12	95.04 ± 0.09	95.75 ± 0.11	74.86 ± 0.48	80.2 ± 0.12	95.05 ± 0.03	86.19 ± 0.25	77.77 ± 0.15	95.88 ± 0.4	88.18
UniOT	**83.66** ± 1.53	95.93 ± 0.71	97.09 ± 0.12	85.3 ± 0.44	94.79 ± 1.04	95.52 ± 0.62	79.53 ± 3.62	81.36 ± 0.86	95.12 ± 0.1	79.36 ± 2.17	82.07 ± 1.19	96.52 ± 0.38	88.85
WiSE-FT	76.68 ± 0.05	94.9 ± 0.03	97.41 ± 0.03	93.97 ± 0.08	**96.57** ± 0.11	97.54 ± 0.04	87.12 ± 0.31	80.76 ± 0.03	96.53 ± 0.02	92.18 ± 0.1	78.95 ± 0.13	96.6 ± 0.01	90.77
CLIP cross-model	74.37 ± 0.12	93.56 ± 0.2	96.71 ± 0.04	92.74 ± 0.18	95.33 ± 0.1	96.82 ± 0.04	86.83 ± 0.01	80.21 ± 0.07	96.41 ± 0.01	89.66 ± 0.05	77.93 ± 0.05	95.92 ± 0.08	89.71
CLIP zero-shot	80.3 ± 0.0	94.82 ± 0.0	94.29 ± 0.0	91.45 ± 0.0	94.82 ± 0.0	94.29 ± 0.0	91.45 ± 0.0	80.3 ± 0.0	94.29 ± 0.0	91.45 ± 0.0	80.3 ± 0.0	94.82 ± 0.0	90.21
Our calibration	83.11 ± 0.0	**96.4** ± 0.0	**97.78** ± 0.0	**94.63** ± 0.0	**96.45** ± 0.0	**97.8** ± 0.0	**94.75** ± 0.0	83.08 ± 0.0	**97.87** ± 0.0	**94.73** ± 0.0	**83.09** ± 0.0	96.59 ± 0.0	**93.02**

**Table A9:** DomainNet: ResNet50 & (150/50) setting. Best results are highlighted in bold.

Methods	P2R	P2S	R2P	R2S	S2P	S2R	Avg
H-score							
SO	42.24 ± 0.13	40.84 ± 0.22	45.31 ± 0.43	41.1 ± 0.12	28.05 ± 0.24	37.67 ± 0.47	39.2
DANCE	20.09 ± 0.26	25.86 ± 0.92	34.6 ± 1.19	41.83 ± 0.42	18.68 ± 0.49	20.42 ± 0.29	26.91
OVANet	55.29 ± 0.08	45.35 ± 0.2	**52.17** ± 0.17	44.69 ± 0.05	44.62 ± 0.33	55.32 ± 0.06	49.57
UniOT	**56.75** ± 0.11	**47.4** ± 0.21	51.72 ± 0.23	**47.23** ± 0.24	**46.17** ± 0.23	**56.02** ± 0.28	**50.88**
H^3^-score							
SO	47.97 ± 0.11	40.92 ± 0.13	47.43 ± 0.29	41.54 ± 0.08	31.99 ± 0.21	43.08 ± 0.39	42.16
DANCE	26.22 ± 0.31	29.49 ± 0.86	38.98 ± 1.04	42.53 ± 0.33	23.42 ± 0.54	26.47 ± 0.31	31.18
OVANet	**58.24** ± 0.07	**44.2** ± 0.15	**52.12** ± 0.12	**43.67** ± 0.08	**46.09** ± 0.23	**57.72** ± 0.1	**50.34**
UniOT	55.7 ± 0.12	42.39 ± 0.18	47.78 ± 0.28	42.51 ± 0.18	44.28 ± 0.17	54.78 ± 0.29	47.91
UCR							
SO	43.03 ± 0.23	25.57 ± 0.21	36.19 ± 0.13	26.03 ± 0.15	21.6 ± 0.02	33.85 ± 0.31	31.04
DANCE	42.56 ± 0.13	23.88 ± 0.58	35.89 ± 0.58	**29.33** ± 0.36	25.61 ± 0.12	38.99 ± 0.09	32.71
OVANet	43.03 ± 0.11	27.57 ± 0.38	**38.18** ± 0.02	27.65 ± 0.26	**30.79** ± 0.29	39.34 ± 0.19	34.43
UniOT	**43.41** ± 0.15	**27.72** ± 0.42	36.16 ± 0.06	28.57 ± 0.34	29.8 ± 0.09	**41.48** ± 0.43	**34.52**

**Table A10:** DomainNet: CLIP & (150/50) setting. Best results are highlighted in bold.

Methods	P2R	P2S	R2P	R2S	S2P	S2R	Avg
H-score							
SO	67.35 ± 0.07	57.55 ± 0.04	58.17 ± 0.12	61.14 ± 0.19	53.01 ± 0.14	71.72 ± 0.06	61.49
DANCE	67.6 ± 0.07	57.33 ± 0.06	55.57 ± 0.25	59.65 ± 0.06	52.09 ± 0.08	70.94 ± 0.12	60.53
OVANet	**74.87** ± 0.15	69.55 ± 0.11	67.97 ± 0.27	70.41 ± 0.17	65.73 ± 0.09	75.65 ± 0.11	70.7
UniOT	74.67 ± 0.39	**69.56** ± 0.19	**69.32** ± 0.47	71.38 ± 0.21	67.15 ± 0.52	**76.42** ± 0.16	71.42
WiSE-FT	5.79 ± 0.04	1.51 ± 0.04	4.24 ± 0.14	3.71 ± 0.03	1.77 ± 0.04	5.45 ± 0.09	3.74
CLIP cross-model	69.19 ± 0.1	58.18 ± 0.14	57.11 ± 0.03	60.85 ± 0.18	53.13 ± 0.16	73.43 ± 0.05	61.98
Our calibration	69.2 ± 0.0	69.09 ± 0.0	**74.78** ± 0.0	**74.85** ± 0.0	**72.68** ± 0.0	73.61 ± 0.0	**72.37**
H^3^-score							
SO	72.52 ± 0.06	58.72 ± 0.03	61.83 ± 0.09	61.17 ± 0.13	57.84 ± 0.11	75.83 ± 0.04	64.65
DANCE	72.71 ± 0.05	58.57 ± 0.04	59.84 ± 0.19	60.16 ± 0.04	57.11 ± 0.06	75.25 ± 0.09	63.94
OVANet	**78.15** ± 0.11	**66.53** ± 0.07	**68.86** ± 0.18	67.06 ± 0.1	67.31 ± 0.06	**78.72** ± 0.08	71.11
UniOT	74.89 ± 0.11	65.78 ± 0.12	67.97 ± 0.34	67.65 ± 0.09	66.15 ± 0.36	76.95 ± 0.25	69.9
WiSE-FT	8.4 ± 0.05	2.24 ± 0.06	6.18 ± 0.2	5.41 ± 0.05	2.63 ± 0.06	7.92 ± 0.13	5.46
CLIP cross-model	73.93 ± 0.08	59.16 ± 0.1	61.03 ± 0.02	60.97 ± 0.12	57.94 ± 0.12	77.1 ± 0.04	65.02
Our calibration	73.94 ± 0.0	66.25 ± 0.0	**73.38** ± 0.0	**69.68** ± 0.0	**72.02** ± 0.0	77.24 ± 0.0	**72.08**
UCR							
SO	66.02 ± 0.08	58.9 ± 0.15	62.84 ± 0.14	62.92 ± 0.1	56.69 ± 0.15	71.78 ± 0.18	63.19
DANCE	67.79 ± 0.1	60.3 ± 0.19	62.77 ± 0.05	63.68 ± 0.09	62.86 ± 0.15	71.88 ± 0.16	64.88
OVANet	68.0 ± 0.2	59.14 ± 0.18	64.61 ± 0.13	62.38 ± 0.11	58.54 ± 0.19	73.13 ± 0.18	64.3
UniOT	68.37 ± 0.31	57.98 ± 0.38	59.43 ± 0.4	61.62 ± 0.05	57.71 ± 0.52	72.16 ± 0.22	62.88
WiSE-FT	73.53 ± 0.11	63.93 ± 0.18	66.78 ± 0.07	66.38 ± 0.1	64.47 ± 0.08	77.24 ± 0.12	68.72
CLIP cross-model	74.17 ± 0.11	63.87 ± 0.05	66.87 ± 0.1	66.39 ± 0.09	63.48 ± 0.19	78.06 ± 0.06	68.81
CLIP zero-shot	79.43 ± 0.0	65.78 ± 0.0	67.12 ± 0.0	65.78 ± 0.0	67.12 ± 0.0	79.43 ± 0.0	70.78
Our calibration	**83.53** ± 0.0	**69.39** ± 0.0	**71.62** ± 0.0	**69.49** ± 0.0	**71.57** ± 0.0	**83.58** ± 0.0	**74.86**

**Table A11:** Office: CLIP & (10/0) setting. Best results are highlighted in bold.

Methods	A2D	A2W	D2A	D2W	W2A	W2D	Avg
H-score							
SO	92.04 ± 0.19	92.01 ± 0.13	91.71 ± 0.27	95.51 ± 0.09	89.77 ± 0.33	90.17 ± 0.43	91.87
DANCE	93.15 ± 0.21	93.08 ± 0.05	**96.74** ± 0.06	**98.74** ± 0.05	**95.81** ± 1.5	**99.0** ± 0.89	96.09
OVANet	93.3 ± 1.17	90.3 ± 0.84	82.93 ± 3.14	97.48 ± 0.11	85.37 ± 0.83	97.58 ± 1.61	91.16
UniOT	**95.11** ± 1.4	**95.92** ± 0.42	95.45 ± 0.71	98.36 ± 1.04	95.68 ± 0.94	98.35 ± 0.17	**96.48**
WiSE-FT	92.77 ± 0.0	90.99 ± 0.0	92.78 ± 0.37	97.87 ± 0.08	92.1 ± 0.23	97.93 ± 0.2	94.07
CLIP cross-model	93.51 ± 0.16	92.19 ± 0.05	93.85 ± 0.01	94.97 ± 0.0	92.47 ± 0.01	94.54 ± 0.08	93.59
Our calibration	89.75 ± 0.0	92.0 ± 0.0	92.67 ± 0.0	93.51 ± 0.0	92.14 ± 0.0	91.28 ± 0.0	91.89
H^3^-score							
SO	91.55 ± 0.12	91.87 ± 0.08	86.0 ± 0.16	94.17 ± 0.06	84.85 ± 0.2	90.32 ± 0.29	89.79
DANCE	92.29 ± 0.14	92.58 ± 0.04	**88.89** ± 0.03	96.24 ± 0.03	**88.36** ± 0.85	96.04 ± 0.56	92.4
OVANet	92.38 ± 0.76	90.73 ± 0.57	80.64 ± 2.0	95.44 ± 0.07	82.18 ± 0.51	95.13 ± 1.03	89.42
UniOT	**94.19** ± 0.85	**95.24** ± 0.09	88.09 ± 0.89	**96.52** ± 0.34	88.22 ± 0.27	**97.18** ± 0.13	**93.24**
WiSE-FT	92.04 ± 0.0	91.19 ± 0.0	86.62 ± 0.22	95.69 ± 0.05	86.22 ± 0.14	95.36 ± 0.13	91.19
CLIP cross-model	92.52 ± 0.1	91.99 ± 0.03	87.24 ± 0.01	93.82 ± 0.0	86.44 ± 0.01	93.19 ± 0.05	90.87
Our calibration	90.04 ± 0.0	91.86 ± 0.0	86.56 ± 0.0	92.86 ± 0.0	86.25 ± 0.0	91.05 ± 0.0	89.77
UCR							
SO	88.49 ± 0.1	91.81 ± 0.21	95.35 ± 0.18	99.32 ± 0.03	94.83 ± 0.18	99.88 ± 0.02	94.95
DANCE	94.74 ± 1.76	94.2 ± 0.04	**97.14** ± 0.01	99.58 ± 0.0	**96.94** ± 0.27	**99.97** ± 0.02	97.09
OVANet	91.89 ± 0.35	94.05 ± 0.29	95.31 ± 0.41	99.42 ± 0.02	94.91 ± 0.22	99.24 ± 1.05	95.8
UniOT	93.27 ± 1.82	97.54 ± 1.0	96.7 ± 0.12	**99.61** ± 0.23	96.65 ± 0.07	99.44 ± 0.4	97.2
WiSE-FT	91.21 ± 0.07	95.19 ± 0.07	96.26 ± 0.09	99.45 ± 0.02	96.13 ± 0.07	99.74 ± 0.3	96.33
CLIP cross-model	90.77 ± 0.27	94.46 ± 0.33	96.53 ± 0.0	99.35 ± 0.0	96.13 ± 0.05	99.85 ± 0.0	96.18
CLIP zero-shot	98.69 ± 0.0	98.98 ± 0.0	95.38 ± 0.0	98.98 ± 0.0	95.38 ± 0.0	98.69 ± 0.0	97.68
Our calibration	**98.74** ± 0.0	**99.0** ± 0.0	95.98 ± 0.0	99.03 ± 0.0	95.98 ± 0.0	98.73 ± 0.0	**97.91**

**Table A12:** OfficeHome: CLIP & (15/0) setting. Best results are highlighted in bold.

Methods	A2C	A2P	A2R	C2A	C2P	C2R	P2A	P2C	P2R	R2A	R2C	R2P	Avg
H-score													
SO	75.41 ± 0.09	83.2 ± 0.05	86.19 ± 0.14	81.69 ± 0.12	87.15 ± 0.21	86.87 ± 0.08	75.93 ± 0.28	76.58 ± 0.06	86.49 ± 0.21	83.02 ± 0.09	78.68 ± 0.02	83.39 ± 0.08	82.05
DANCE	80.03 ± 0.04	89.48 ± 0.04	87.88 ± 0.1	80.8 ± 0.1	89.69 ± 0.17	88.31 ± 0.09	73.42 ± 0.06	76.28 ± 0.14	86.18 ± 0.16	83.27 ± 0.04	80.29 ± 0.05	91.72 ± 0.25	83.95
OVANet	78.42 ± 0.21	84.47 ± 0.43	88.3 ± 0.27	80.71 ± 0.41	84.84 ± 0.45	86.53 ± 0.11	64.85 ± 0.7	60.25 ± 0.53	84.74 ± 0.22	82.66 ± 0.17	77.01 ± 0.2	90.66 ± 0.49	80.29
UniOT	**84.19** ± 0.27	**90.91** ± 0.95	**90.48** ± 0.58	83.97 ± 0.52	**91.71** ± 0.97	88.47 ± 0.82	80.06 ± 1.1	**82.36** ± 0.62	87.92 ± 0.26	85.26 ± 0.45	**82.58** ± 0.95	**91.76** ± 0.63	**86.64**
WiSE-FT	64.63 ± 0.04	82.12 ± 0.16	84.11 ± 0.03	60.82 ± 0.31	82.93 ± 0.25	79.64 ± 0.26	59.95 ± 0.38	54.5 ± 0.31	80.59 ± 0.1	71.29 ± 0.23	69.77 ± 0.27	90.9 ± 0.22	73.44
CLIP cross-model	77.8 ± 0.06	84.46 ± 0.06	87.64 ± 0.08	84.68 ± 0.1	87.19 ± 0.23	**88.59** ± 0.09	81.77 ± 0.11	81.85 ± 0.1	89.5 ± 0.09	84.61 ± 0.09	79.31 ± 0.15	83.66 ± 0.1	84.26
Our calibration	80.99 ± 0.0	78.58 ± 0.0	84.97 ± 0.0	**87.66** ± 0.0	81.09 ± 0.0	86.76 ± 0.0	**86.46** ± 0.0	81.5 ± 0.0	**92.56** ± 0.0	**87.44** ± 0.0	82.25 ± 0.0	86.96 ± 0.0	84.77
H^3^-score													
SO	74.74 ± 0.06	85.58 ± 0.04	85.83 ± 0.09	77.2 ± 0.07	88.32 ± 0.14	86.28 ± 0.05	73.68 ± 0.17	75.51 ± 0.04	86.03 ± 0.14	77.99 ± 0.05	76.86 ± 0.02	85.71 ± 0.06	81.14
DANCE	77.71 ± 0.02	89.91 ± 0.02	86.95 ± 0.06	76.67 ± 0.06	90.04 ± 0.12	87.22 ± 0.06	72.09 ± 0.04	75.31 ± 0.09	85.82 ± 0.11	78.13 ± 0.02	77.87 ± 0.03	91.4 ± 0.17	82.43
OVANet	76.69 ± 0.13	86.47 ± 0.3	87.22 ± 0.18	76.61 ± 0.24	86.73 ± 0.31	86.06 ± 0.07	66.34 ± 0.49	64.09 ± 0.4	84.87 ± 0.15	77.77 ± 0.1	75.78 ± 0.13	90.69 ± 0.33	79.94
UniOT	**80.45** ± 0.38	**91.01** ± 0.54	**89.65** ± 0.24	**80.66** ± 0.8	**91.68** ± 0.64	**87.73** ± 0.51	78.7 ± 0.74	**79.83** ± 0.34	87.94 ± 0.34	**82.5** ± 0.48	**79.68** ± 0.48	**92.04** ± 0.43	**85.16**
WiSE-FT	67.32 ± 0.03	84.82 ± 0.11	84.45 ± 0.02	63.48 ± 0.22	85.39 ± 0.18	81.39 ± 0.18	62.84 ± 0.28	59.62 ± 0.24	82.05 ± 0.07	70.7 ± 0.15	70.96 ± 0.19	90.86 ± 0.15	75.32
CLIP cross-model	76.29 ± 0.04	86.47 ± 0.04	86.79 ± 0.06	78.96 ± 0.06	88.35 ± 0.16	87.41 ± 0.06	77.25 ± 0.06	78.84 ± 0.06	88.0 ± 0.06	78.92 ± 0.05	77.26 ± 0.09	85.9 ± 0.07	82.54
Our calibration	78.31 ± 0.0	82.26 ± 0.0	85.02 ± 0.0	80.66 ± 0.0	84.08 ± 0.0	86.21 ± 0.0	**79.98** ± 0.0	78.63 ± 0.0	**89.94** ± 0.0	80.54 ± 0.0	79.09 ± 0.0	88.19 ± 0.0	82.74
UCR													
SO	73.39 ± 0.06	89.19 ± 0.07	92.23 ± 0.02	87.93 ± 0.05	92.11 ± 0.15	90.87 ± 0.18	72.67 ± 0.26	74.93 ± 0.13	89.21 ± 0.07	81.48 ± 0.04	76.85 ± 0.16	93.48 ± 0.07	84.53
DANCE	80.41 ± 0.04	91.01 ± 0.22	92.63 ± 0.08	88.15 ± 0.09	93.8 ± 0.05	91.57 ± 0.1	75.77 ± 0.02	78.93 ± 0.19	91.13 ± 0.28	83.35 ± 0.21	80.17 ± 0.1	94.22 ± 0.05	86.76
OVANet	76.65 ± 0.18	92.89 ± 0.06	93.55 ± 0.15	87.56 ± 0.1	93.41 ± 0.2	90.89 ± 0.26	71.32 ± 0.23	77.95 ± 0.06	90.35 ± 0.07	81.78 ± 0.22	78.15 ± 0.1	94.09 ± 0.34	85.72
UniOT	79.4 ± 0.54	90.87 ± 2.05	94.6 ± 0.51	83.25 ± 0.88	93.25 ± 0.34	90.9 ± 0.92	76.83 ± 4.88	75.45 ± 1.72	89.5 ± 0.32	80.12 ± 1.47	78.79 ± 0.85	94.17 ± 0.73	85.59
WiSE-FT	78.15 ± 0.02	93.32 ± 0.04	**95.79** ± 0.03	92.46 ± 0.05	**95.31** ± 0.15	95.16 ± 0.06	83.67 ± 0.19	80.21 ± 0.02	93.99 ± 0.06	88.57 ± 0.13	79.71 ± 0.09	**95.0** ± 0.0	89.28
CLIP cross-model	75.95 ± 0.1	91.07 ± 0.13	93.99 ± 0.06	90.54 ± 0.18	93.73 ± 0.1	93.36 ± 0.09	83.61 ± 0.07	79.94 ± 0.09	93.65 ± 0.08	85.53 ± 0.08	78.29 ± 0.04	94.15 ± 0.03	87.82
CLIP zero-shot	80.42 ± 0.0	93.53 ± 0.0	93.71 ± 0.0	91.03 ± 0.0	93.53 ± 0.0	93.71 ± 0.0	91.03 ± 0.0	80.42 ± 0.0	93.71 ± 0.0	91.03 ± 0.0	80.42 ± 0.0	93.53 ± 0.0	89.67
Our calibration	**82.27** ± 0.0	**94.52** ± 0.0	**96.52** ± 0.0	**93.32** ± 0.0	94.59 ± 0.0	**96.56** ± 0.0	**93.48** ± 0.0	**82.29** ± 0.0	**96.71** ± 0.0	**93.45** ± 0.0	**82.29** ± 0.0	94.81 ± 0.0	**91.73**

**Table A13:** DomainNet: CLIP & (150/0) setting. Best results are highlighted in bold.

Methods	P2R	P2S	R2P	R2S	S2P	S2R	Avg
H-score							
SO	72.15 ± 0.06	62.77 ± 0.1	62.5 ± 0.19	65.55 ± 0.09	56.61 ± 0.05	74.19 ± 0.03	65.63
DANCE	72.2 ± 0.07	62.16 ± 0.2	60.81 ± 0.06	65.86 ± 0.16	56.69 ± 0.01	73.75 ± 0.02	65.25
OVANet	**77.09** ± 0.17	**71.64** ± 0.04	69.57 ± 0.13	72.51 ± 0.1	67.04 ± 0.11	76.58 ± 0.11	72.41
UniOT	76.78 ± 0.08	70.35 ± 0.31	71.84 ± 0.05	73.56 ± 0.15	69.32 ± 0.36	**77.43** ± 0.19	73.21
WiSE-FT	11.76 ± 0.11	4.52 ± 0.09	8.84 ± 0.11	7.73 ± 0.07	3.77 ± 0.08	10.9 ± 0.13	7.92
CLIP cross-model	74.9 ± 0.1	64.16 ± 0.03	62.16 ± 0.06	65.96 ± 0.05	58.29 ± 0.04	77.11 ± 0.08	67.1
Our calibration	71.59 ± 0.0	70.67 ± 0.0	**76.39** ± 0.0	**76.81** ± 0.0	**73.88** ± 0.0	76.01 ± 0.0	**74.22**
H^3^-score							
SO	75.38 ± 0.04	63.2 ± 0.07	64.39 ± 0.14	65.05 ± 0.06	60.09 ± 0.04	76.86 ± 0.02	67.5
DANCE	75.42 ± 0.05	62.78 ± 0.14	63.18 ± 0.04	65.25 ± 0.11	60.15 ± 0.01	76.54 ± 0.02	67.22
OVANet	**78.9** ± 0.12	**68.92** ± 0.03	69.22 ± 0.09	69.46 ± 0.06	67.53 ± 0.07	78.55 ± 0.08	72.1
UniOT	76.08 ± 0.08	67.15 ± 0.42	68.52 ± 0.1	69.74 ± 0.09	66.52 ± 0.07	76.99 ± 0.19	70.83
WiSE-FT	16.48 ± 0.15	6.55 ± 0.13	12.46 ± 0.15	10.93 ± 0.1	5.51 ± 0.12	15.34 ± 0.17	11.21
CLIP cross-model	77.36 ± 0.07	64.13 ± 0.02	64.15 ± 0.04	65.32 ± 0.03	61.35 ± 0.03	**78.92** ± 0.05	68.54
Our calibration	74.97 ± 0.0	68.32 ± 0.0	**73.58** ± 0.0	**72.04** ± 0.0	**72.01** ± 0.0	78.15 ± 0.0	**73.18**
UCR							
SO	70.57 ± 0.02	62.09 ± 0.09	64.72 ± 0.15	65.34 ± 0.17	59.2 ± 0.21	74.13 ± 0.04	66.01
DANCE	72.14 ± 0.06	63.86 ± 0.13	65.17 ± 0.13	67.27 ± 0.02	66.4 ± 0.05	75.36 ± 0.03	68.37
OVANet	72.32 ± 0.16	62.88 ± 0.06	65.72 ± 0.18	65.36 ± 0.02	60.15 ± 0.14	75.22 ± 0.1	66.94
UniOT	73.72 ± 0.17	61.89 ± 0.29	64.05 ± 0.18	65.77 ± 0.19	62.91 ± 0.19	75.66 ± 0.22	67.33
WiSE-FT	77.55 ± 0.03	67.24 ± 0.08	68.76 ± 0.1	68.97 ± 0.16	67.45 ± 0.11	79.97 ± 0.02	71.66
CLIP cross-model	78.0 ± 0.09	67.6 ± 0.15	68.62 ± 0.16	68.86 ± 0.05	65.94 ± 0.11	80.14 ± 0.08	71.53
CLIP zero-shot	82.0 ± 0.0	68.37 ± 0.0	69.64 ± 0.0	68.37 ± 0.0	69.64 ± 0.0	82.0 ± 0.0	73.34
Our calibration	**85.72** ± 0.0	**71.97** ± 0.0	**73.89** ± 0.0	**72.07** ± 0.0	**73.84** ± 0.0	**85.78** ± 0.0	**77.21**

**Table A14:** Office: CLIP & (31/0) setting. Best results are highlighted in bold.

Methods	A2D	A2W	D2A	D2W	W2A	W2D	Avg
H-score/H^3^-score							
SO	76.99 ± 0.54	71.98 ± 0.1	68.54 ± 0.19	97.64 ± 0.15	66.94 ± 0.25	99.26 ± 0.0	80.22
DANCE	73.29 ± 1.1	63.14 ± 0.16	62.46 ± 1.66	**98.4** ± 0.0	57.63 ± 3.83	**99.64** ± 0.32	75.76
OVANet	72.5 ± 0.73	64.35 ± 0.3	58.02 ± 1.13	96.26 ± 0.05	58.07 ± 0.66	98.67 ± 0.0	74.64
UniOT	58.03 ± 2.06	56.8 ± 1.14	53.8 ± 0.89	69.88 ± 1.5	53.23 ± 0.88	67.96 ± 3.44	59.95
WiSE-FT	31.89 ± 0.31	25.87 ± 0.19	39.04 ± 0.14	74.62 ± 0.26	36.65 ± 0.29	79.13 ± 0.09	47.87
CLIP cross-model	79.87 ± 0.45	77.27 ± 0.38	73.99 ± 0.08	97.46 ± 0.0	72.03 ± 0.05	98.65 ± 0.12	83.21
Our calibration	**82.52** ± 0.0	**82.41** ± 0.0	**82.91** ± 0.0	85.71 ± 0.0	**83.25** ± 0.0	85.97 ± 0.0	**83.8**
UCR							
SO	93.37 ± 0.28	**95.14** ± 0.26	81.03 ± 0.08	99.66 ± 0.06	79.65 ± 0.09	99.8 ± 0.0	91.44
DANCE	89.56 ± 0.28	85.7 ± 0.57	76.3 ± 0.32	**99.75** ± 0.0	74.81 ± 0.92	**100.0** ± 0.0	87.69
OVANet	93.31 ± 0.25	94.93 ± 0.16	81.04 ± 0.09	99.66 ± 0.06	79.64 ± 0.15	99.8 ± 0.0	91.4
UniOT	92.44 ± 0.96	94.17 ± 0.99	84.17 ± 0.56	98.78 ± 0.62	84.32 ± 0.3	98.93 ± 0.34	92.14
WiSE-FT	93.24 ± 0.19	92.91 ± 0.42	84.76 ± 0.07	98.99 ± 0.0	84.2 ± 0.1	99.67 ± 0.09	92.3
CLIP cross-model	**94.98** ± 0.0	94.8 ± 0.16	85.56 ± 0.04	99.75 ± 0.0	84.52 ± 0.09	99.8 ± 0.0	**93.23**
CLIP zero-shot	88.15 ± 0.0	89.18 ± 0.0	85.73 ± 0.0	89.18 ± 0.0	85.73 ± 0.0	88.15 ± 0.0	87.69
Our calibration	88.15 ± 0.0	89.18 ± 0.0	**85.73** ± 0.0	89.18 ± 0.0	**85.73** ± 0.0	88.15 ± 0.0	87.69

**Table A15:** OfficeHome: CLIP & (65/0) setting. Best results are highlighted in bold.

Methods	A2C	A2P	A2R	C2A	C2P	C2R	P2A	P2C	P2R	R2A	R2C	R2P	Avg
H-score/H^3^-score													
SO	42.18 ± 0.02	65.78 ± 0.1	67.64 ± 0.01	46.06 ± 0.18	68.16 ± 0.16	64.49 ± 0.08	42.68 ± 0.12	37.5 ± 0.07	71.0 ± 0.07	58.19 ± 0.19	48.05 ± 0.09	85.69 ± 0.05	58.12
DANCE	39.45 ± 0.12	59.51 ± 1.16	68.27 ± 0.05	45.45 ± 0.17	58.08 ± 0.31	63.0 ± 0.1	36.2 ± 0.56	35.31 ± 0.1	68.49 ± 0.29	58.1 ± 0.47	47.07 ± 0.06	86.12 ± 0.02	55.42
OVANet	53.14 ± 0.16	67.63 ± 0.26	77.34 ± 0.07	61.55 ± 0.37	67.28 ± 0.31	73.51 ± 0.21	50.26 ± 0.2	40.58 ± 0.43	75.23 ± 0.19	68.72 ± 0.14	53.28 ± 0.23	87.32 ± 0.2	64.65
UniOT	52.4 ± 0.23	54.54 ± 0.9	64.91 ± 0.76	61.16 ± 0.31	66.6 ± 1.57	72.71 ± 0.73	47.04 ± 0.66	47.5 ± 0.23	59.05 ± 0.61	60.14 ± 0.58	54.32 ± 0.22	70.81 ± 1.06	59.27
WiSE-FT	5.98 ± 0.11	14.86 ± 0.05	17.47 ± 0.04	3.65 ± 0.12	9.79 ± 0.11	9.0 ± 0.13	9.35 ± 0.07	5.21 ± 0.08	26.2 ± 0.02	14.31 ± 0.11	8.7 ± 0.18	39.13 ± 0.07	13.64
CLIP cross-model	44.97 ± 0.19	73.26 ± 0.06	72.75 ± 0.08	52.76 ± 0.25	77.72 ± 0.06	71.95 ± 0.09	47.85 ± 0.1	41.82 ± 0.04	73.98 ± 0.09	58.98 ± 0.03	49.1 ± 0.09	86.71 ± 0.03	62.65
Our calibration	**69.65** ± 0.0	**92.95** ± 0.0	**92.37** ± 0.0	**81.08** ± 0.0	**92.65** ± 0.0	**91.97** ± 0.0	**70.84** ± 0.0	**59.19** ± 0.0	**86.44** ± 0.0	**75.15** ± 0.0	**63.08** ± 0.0	**91.53** ± 0.0	**80.58**
UCR													
SO	72.07 ± 0.11	87.95 ± 0.04	90.96 ± 0.04	82.19 ± 0.19	88.87 ± 0.06	89.79 ± 0.08	77.21 ± 0.15	69.28 ± 0.27	89.85 ± 0.09	85.55 ± 0.02	75.01 ± 0.05	93.86 ± 0.07	83.55
DANCE	71.78 ± 0.19	82.62 ± 0.23	90.46 ± 0.01	81.33 ± 0.17	86.07 ± 0.11	88.24 ± 0.15	72.35 ± 0.34	69.52 ± 0.17	87.54 ± 0.08	84.74 ± 0.1	74.08 ± 0.08	92.16 ± 0.08	81.74
OVANet	72.07 ± 0.12	87.99 ± 0.08	90.98 ± 0.06	82.01 ± 0.14	88.78 ± 0.14	89.79 ± 0.05	77.27 ± 0.07	69.14 ± 0.09	89.75 ± 0.08	85.51 ± 0.16	75.22 ± 0.3	93.77 ± 0.2	83.52
UniOT	75.48 ± 0.86	91.03 ± 0.24	92.17 ± 0.23	84.97 ± 0.91	90.74 ± 0.41	90.18 ± 0.09	79.06 ± 1.0	73.81 ± 0.14	90.98 ± 0.13	85.87 ± 0.29	**77.72** ± 0.3	93.97 ± 0.39	85.5
WiSE-FT	76.3 ± 0.08	92.93 ± 0.03	93.61 ± 0.03	87.76 ± 0.09	92.36 ± 0.07	93.02 ± 0.02	82.98 ± 0.12	74.78 ± 0.08	92.4 ± 0.04	88.74 ± 0.19	77.6 ± 0.08	94.86 ± 0.02	87.28
CLIP cross-model	75.67 ± 0.08	93.52 ± 0.07	93.33 ± 0.01	86.24 ± 0.09	92.14 ± 0.02	92.33 ± 0.1	82.75 ± 0.07	74.43 ± 0.12	92.39 ± 0.05	88.31 ± 0.13	77.15 ± 0.12	**95.17** ± 0.01	86.95
CLIP zero-shot	77.69 ± 0.0	94.32 ± 0.0	94.51 ± 0.0	89.82 ± 0.0	94.32 ± 0.0	94.51 ± 0.0	89.82 ± 0.0	77.69 ± 0.0	94.51 ± 0.0	89.82 ± 0.0	77.69 ± 0.0	94.32 ± 0.0	89.08
Our calibration	**77.69** ± 0.0	**94.32** ± 0.0	**94.51** ± 0.0	**89.82** ± 0.0	**94.32** ± 0.0	**94.51** ± 0.0	**89.82** ± 0.0	**77.69** ± 0.0	**94.51** ± 0.0	**89.82** ± 0.0	77.69 ± 0.0	94.32 ± 0.0	**89.08**

**Table A16:** DomainNet: CLIP & (345/0) setting. Best results are highlighted in bold.

Methods	P2R	P2S	R2P	R2S	S2P	S2R	Avg
H-score/H^3^-score							
SO	47.32 ± 0.07	31.92 ± 0.03	33.24 ± 0.06	35.42 ± 0.06	30.24 ± 0.04	51.46 ± 0.04	38.27
DANCE	47.09 ± 0.03	32.1 ± 0.11	31.62 ± 0.24	33.88 ± 0.14	29.89 ± 0.11	50.78 ± 0.04	37.56
OVANet	65.92 ± 0.1	51.21 ± 0.11	49.47 ± 0.07	54.56 ± 0.05	51.87 ± 0.19	70.27 ± 0.09	57.22
UniOT	71.17 ± 0.32	60.36 ± 0.17	57.09 ± 0.35	63.79 ± 0.17	56.98 ± 0.15	72.93 ± 0.07	63.72
WiSE-FT	0.56 ± 0.01	0.09 ± 0.0	0.47 ± 0.03	0.25 ± 0.01	0.11 ± 0.01	0.3 ± 0.01	0.3
CLIP cross-model	46.33 ± 0.11	29.96 ± 0.09	29.69 ± 0.12	32.42 ± 0.1	28.35 ± 0.06	50.46 ± 0.06	36.2
Our calibration	**85.82** ± 0.0	**72.1** ± 0.0	**66.93** ± 0.0	**70.96** ± 0.0	**67.97** ± 0.0	**85.78** ± 0.0	**74.93**
UCR							
SO	76.06 ± 0.08	64.06 ± 0.23	69.55 ± 0.18	68.6 ± 0.04	67.67 ± 0.13	81.64 ± 0.08	71.26
DANCE	75.6 ± 0.11	66.47 ± 0.13	70.01 ± 0.03	68.07 ± 0.03	69.35 ± 0.12	80.44 ± 0.06	71.66
OVANet	76.15 ± 0.06	64.36 ± 0.23	69.65 ± 0.09	68.78 ± 0.05	67.66 ± 0.14	81.63 ± 0.05	71.37
UniOT	79.76 ± 0.04	68.2 ± 0.13	71.57 ± 0.2	71.12 ± 0.1	69.99 ± 0.04	82.45 ± 0.08	73.85
WiSE-FT	82.2 ± 0.08	69.95 ± 0.11	72.67 ± 0.08	71.79 ± 0.02	72.33 ± 0.11	85.51 ± 0.02	75.74
CLIP cross-model	82.4 ± 0.01	69.93 ± 0.16	72.48 ± 0.1	71.33 ± 0.02	71.61 ± 0.06	85.66 ± 0.04	75.57
CLIP zero-shot	88.5 ± 0.0	74.54 ± 0.0	75.27 ± 0.0	74.65 ± 0.0	75.27 ± 0.0	88.68 ± 0.0	79.48
Our calibration	**88.5** ± 0.0	**74.54** ± 0.0	**75.27** ± 0.0	**74.66** ± 0.0	**75.27** ± 0.0	**88.68** ± 0.0	**79.49**

**Table A17:** Office: CLIP & (10/21) setting. Best results are highlighted in bold.

Methods	A2D	A2W	D2A	D2W	W2A	W2D	Avg
H-score/H^3^-score							
SO	86.4 ± 0.5	83.72 ± 0.24	89.13 ± 0.52	96.43 ± 0.22	84.28 ± 0.55	**98.75** ± 0.0	89.79
DANCE	65.23 ± 6.21	43.33 ± 4.71	57.13 ± 8.0	90.0 ± 0.0	47.77 ± 7.84	97.5 ± 0.0	66.83
OVANet	91.42 ± 0.2	77.48 ± 2.74	81.96 ± 4.4	95.79 ± 0.11	81.63 ± 4.31	96.92 ± 0.0	87.53
UniOT	43.36 ± 1.88	36.25 ± 2.27	39.29 ± 1.02	42.39 ± 0.83	37.23 ± 2.93	49.34 ± 1.09	41.31
WiSE-FT	39.89 ± 0.39	28.34 ± 0.15	50.65 ± 0.36	76.36 ± 0.18	43.01 ± 0.44	83.15 ± 0.39	53.57
CLIP cross-model	89.62 ± 0.2	87.18 ± 0.18	93.65 ± 0.13	**96.64** ± 0.0	91.03 ± 0.13	97.19 ± 0.2	92.55
Our calibration	**93.32** ± 0.0	**91.33** ± 0.0	**95.81** ± 0.0	94.81 ± 0.0	**96.01** ± 0.0	95.05 ± 0.0	**94.39**
UCR							
SO	95.12 ± 0.6	97.63 ± 0.28	95.27 ± 0.05	99.44 ± 0.16	94.5 ± 0.13	**100.0** ± 0.0	96.99
DANCE	76.01 ± 5.34	71.41 ± 1.31	76.55 ± 2.69	91.53 ± 0.0	71.71 ± 3.41	99.36 ± 0.0	81.09
OVANet	94.69 ± 0.6	97.18 ± 0.16	95.27 ± 0.05	99.44 ± 0.16	94.43 ± 0.18	100.0 ± 0.0	96.83
UniOT	47.56 ± 2.1	48.14 ± 1.73	61.97 ± 0.63	63.73 ± 2.0	58.21 ± 1.8	56.05 ± 0.52	55.94
WiSE-FT	**96.18** ± 0.0	**97.74** ± 0.16	96.31 ± 0.05	**99.66** ± 0.0	95.93 ± 0.09	99.58 ± 0.3	97.57
CLIP cross-model	96.18 ± 0.0	97.63 ± 0.28	96.35 ± 0.0	99.66 ± 0.0	95.65 ± 0.05	100.0 ± 0.0	**97.58**
CLIP zero-shot	95.54 ± 0.0	97.63 ± 0.0	**96.66** ± 0.0	97.63 ± 0.0	**96.66** ± 0.0	95.54 ± 0.0	96.61
Our calibration	95.54 ± 0.0	97.63 ± 0.0	**96.66** ± 0.0	97.63 ± 0.0	**96.66** ± 0.0	95.54 ± 0.0	96.61

**Table A18:** OfficeHome: CLIP & (25/40) setting. Best results are highlighted in bold.

Methods	A2C	A2P	A2R	C2A	C2P	C2R	P2A	P2C	P2R	R2A	R2C	R2P	Avg
H-score/H^3^-score													
SO	44.33 ± 0.19	65.34 ± 0.35	71.4 ± 0.35	44.78 ± 0.1	59.45 ± 0.47	63.91 ± 0.17	46.61 ± 0.26	41.37 ± 0.14	72.02 ± 0.12	59.08 ± 0.17	53.28 ± 0.1	78.2 ± 0.12	58.31
DANCE	34.87 ± 0.17	41.08 ± 1.78	74.03 ± 1.62	31.93 ± 1.16	34.31 ± 0.22	54.84 ± 2.92	25.85 ± 1.81	21.6 ± 0.81	63.45 ± 2.65	57.55 ± 0.35	45.7 ± 0.79	74.36 ± 0.06	46.63
OVANet	60.09 ± 0.43	70.91 ± 0.3	81.93 ± 0.39	60.59 ± 0.48	61.88 ± 1.41	72.19 ± 0.26	52.46 ± 0.48	45.8 ± 1.0	76.8 ± 0.16	68.38 ± 0.23	58.47 ± 0.2	81.48 ± 0.52	65.92
UniOT	44.17 ± 0.19	43.91 ± 0.93	39.98 ± 1.07	47.21 ± 0.58	41.66 ± 2.14	47.94 ± 3.9	37.16 ± 1.5	44.8 ± 0.48	39.38 ± 1.05	48.13 ± 0.6	43.92 ± 0.44	44.92 ± 2.23	43.6
WiSE-FT	9.8 ± 0.04	17.69 ± 0.16	22.75 ± 0.08	6.1 ± 0.04	11.27 ± 0.08	13.4 ± 0.28	11.26 ± 0.09	7.26 ± 0.14	29.79 ± 0.09	17.6 ± 0.12	13.3 ± 0.28	38.55 ± 0.19	16.56
CLIP cross-model	48.18 ± 0.04	70.76 ± 0.25	75.41 ± 0.24	51.32 ± 0.17	70.49 ± 0.36	70.68 ± 0.13	52.32 ± 0.19	48.38 ± 0.12	75.23 ± 0.09	59.69 ± 0.2	55.33 ± 0.29	79.92 ± 0.18	63.14
Our calibration	**73.32** ± 0.0	**90.08** ± 0.0	**92.55** ± 0.0	**81.06** ± 0.0	**89.85** ± 0.0	**92.22** ± 0.0	**70.63** ± 0.0	**63.18** ± 0.0	**87.26** ± 0.0	**74.48** ± 0.0	**66.75** ± 0.0	**88.36** ± 0.0	**80.81**
UCR													
SO	79.38 ± 0.32	87.73 ± 0.24	91.94 ± 0.14	81.14 ± 0.38	81.94 ± 0.21	87.7 ± 0.33	78.6 ± 0.3	76.66 ± 0.22	91.33 ± 0.09	84.82 ± 0.16	81.93 ± 0.16	90.05 ± 0.1	84.44
DANCE	69.39 ± 0.34	75.16 ± 1.63	90.8 ± 0.4	72.39 ± 0.6	70.55 ± 0.52	82.33 ± 0.83	62.63 ± 0.81	61.95 ± 0.7	83.45 ± 0.18	80.38 ± 0.11	74.07 ± 0.35	84.48 ± 0.09	75.63
OVANet	79.5 ± 0.12	87.45 ± 0.12	91.81 ± 0.09	80.96 ± 0.3	81.89 ± 0.03	87.61 ± 0.29	78.51 ± 0.0	76.42 ± 0.15	91.22 ± 0.08	84.73 ± 0.17	82.41 ± 0.2	89.9 ± 0.44	84.37
UniOT	57.03 ± 1.24	58.43 ± 0.76	75.43 ± 1.72	64.19 ± 3.19	68.33 ± 2.08	79.72 ± 2.56	44.14 ± 0.74	55.84 ± 1.23	69.87 ± 1.01	63.7 ± 0.87	59.86 ± 0.61	66.76 ± 0.11	63.61
WiSE-FT	**82.93** ± 0.21	91.69 ± 0.19	93.82 ± 0.12	88.03 ± 0.23	87.58 ± 0.23	91.9 ± 0.13	84.21 ± 0.2	82.07 ± 0.16	93.34 ± 0.03	88.77 ± 0.35	**84.82** ± 0.14	92.12 ± 0.03	88.44
CLIP cross-model	82.37 ± 0.15	**92.57** ± 0.12	93.12 ± 0.16	85.8 ± 0.09	86.87 ± 0.1	90.34 ± 0.05	84.27 ± 0.04	81.83 ± 0.32	93.58 ± 0.03	87.82 ± 0.09	84.3 ± 0.05	**92.75** ± 0.12	87.97
CLIP zero-shot	81.61 ± 0.0	91.2 ± 0.0	94.53 ± 0.0	90.36 ± 0.0	91.2 ± 0.0	94.53 ± 0.0	90.36 ± 0.0	81.61 ± 0.0	94.53 ± 0.0	90.36 ± 0.0	81.61 ± 0.0	91.2 ± 0.0	89.43
Our calibration	81.61 ± 0.0	91.2 ± 0.0	**94.53** ± 0.0	**90.36** ± 0.0	**91.2** ± 0.0	**94.53** ± 0.0	**90.36** ± 0.0	81.61 ± 0.0	**94.53** ± 0.0	**90.36** ± 0.0	81.61 ± 0.0	91.2 ± 0.0	**89.43**

**Table A19:** DomainNet: CLIP & (150/195) setting. Best results are highlighted in bold.

Methods	P2R	P2S	R2P	R2S	S2P	S2R	Avg
H-score/H^3^-score							
SO	44.52 ± 0.09	31.86 ± 0.16	32.39 ± 0.02	34.58 ± 0.12	25.71 ± 0.11	46.24 ± 0.26	35.88
DANCE	42.13 ± 0.74	28.66 ± 0.23	25.54 ± 0.33	27.51 ± 0.41	21.75 ± 0.42	39.93 ± 0.71	30.92
OVANet	65.01 ± 0.07	51.31 ± 0.26	48.81 ± 0.22	54.76 ± 0.22	47.84 ± 0.25	67.4 ± 0.19	55.85
UniOT	60.68 ± 0.59	54.95 ± 0.29	50.63 ± 0.31	56.8 ± 0.05	47.35 ± 0.97	60.69 ± 0.56	55.18
WiSE-FT	0.57 ± 0.01	0.06 ± 0.01	0.52 ± 0.04	0.32 ± 0.02	0.07 ± 0.01	0.2 ± 0.01	0.29
CLIP cross-model	43.44 ± 0.12	29.73 ± 0.18	28.59 ± 0.05	31.68 ± 0.23	24.92 ± 0.05	46.01 ± 0.03	34.06
Our calibration	**85.69** ± 0.0	**73.22** ± 0.0	**67.02** ± 0.0	**71.65** ± 0.0	**68.36** ± 0.0	**85.4** ± 0.0	**75.22**
UCR							
SO	74.92 ± 0.05	63.6 ± 0.4	68.76 ± 0.24	70.77 ± 0.11	67.52 ± 0.11	79.14 ± 0.15	70.78
DANCE	72.51 ± 0.15	63.94 ± 0.37	64.22 ± 0.24	64.88 ± 0.31	64.98 ± 0.06	75.05 ± 0.17	67.6
OVANet	74.9 ± 0.05	64.19 ± 0.25	68.84 ± 0.13	71.06 ± 0.07	67.52 ± 0.27	79.11 ± 0.08	70.94
UniOT	75.12 ± 0.17	64.5 ± 0.34	64.31 ± 0.24	67.24 ± 0.32	61.14 ± 0.89	75.63 ± 0.27	67.99
WiSE-FT	81.23 ± 0.08	70.46 ± 0.23	72.08 ± 0.11	74.16 ± 0.09	72.95 ± 0.09	83.74 ± 0.02	75.77
CLIP cross-model	81.34 ± 0.08	70.44 ± 0.21	72.06 ± 0.14	73.51 ± 0.06	71.97 ± 0.19	84.0 ± 0.11	75.55
CLIP zero-shot	87.99 ± 0.0	76.44 ± 0.0	75.2 ± 0.0	76.41 ± 0.0	75.2 ± 0.0	87.98 ± 0.0	79.87
Our calibration	**88.0** ± 0.0	**76.44** ± 0.0	**75.2** ± 0.0	**76.41** ± 0.0	**75.2** ± 0.0	**87.99** ± 0.0	**79.87**
